# A machine learning based prediction system for the Indian Ocean Dipole

**DOI:** 10.1038/s41598-019-57162-8

**Published:** 2020-01-14

**Authors:** J. V. Ratnam, H. A. Dijkstra, Swadhin K. Behera

**Affiliations:** 1 Application Laboratory, Japan Agency for Marine-Earth Science and Technology, Yokohama, Japan; 2 Institute for Marine and Atmospheric research Utrecht, Utrecht University, Utrecht, The Netherlands

**Keywords:** Atmospheric dynamics, Climate and Earth system modelling

## Abstract

The Indian Ocean Dipole (IOD) is a mode of climate variability observed in the Indian Ocean sea surface temperature anomalies with one pole off Sumatra and the other pole near East Africa. An IOD event starts sometime in May-June, peaks in September-October and ends in November. Through atmospheric teleconnections, it affects the climate of many parts of the world, especially that of East Africa, Australia, India, Japan, and Europe. Owing to its large impacts, previous studies have addressed the predictability of the IOD using state of the art coupled climate models. Here, for the first-time, we predict the IOD using machine learning techniques, in particular artificial neural networks (ANNs). The IOD forecasts are generated for May to November from February-April conditions. The attributes for the ANNs are derived from sea surface temperature, 850 hPa and 200 hPa geopotential height anomalies, using a correlation analysis for the period 1949–2018. An ensemble of ANN forecasts is generated using 500 samples with replacement using jackknife approach. The ensemble mean of the IOD forecasts indicates the machine learning based ANN models to be capable of forecasting the IOD index well in advance with excellent skills. The forecast skills are much superior to the skills obtained from the persistence forecasts that one would guess from the observed data. The ANN models also perform far better than the models of the North American Multi-Model Ensemble (NMME) with higher correlation coefficients and lower root mean square errors (RMSE) for all the target months of May-November.

## Introduction

The Indian Ocean Dipole (IOD)^1–4^ is the dominant mode of climate variability in the Indian Ocean. The positive (negative) phase of IOD is characterized by negative (positive) SST anomalies off Sumatra and positive (negative) SST anomalies near East Africa. Therefore, the IOD is represented by an index derived from the gradient between the western equatorial Indian Ocean and the south eastern equatorial Indian Ocean. It starts sometime in May-June and ends in November and is known to affect the climates of many parts of the world, e.g. that of Australia, India, Sri Lanka, Maritime Continent and East Africa through atmospheric teleconnections. In recent years, it is found that the spatial distribution of the summer rainfall over India is affected by IOD during its various phases^2–4^. During the positive IOD phase, India experiences anomalously high rainfall along the latitude belts covering central India and during the negative phase of the IOD the rainfall is anomalously high along the longitudinal belt with the western part receiving high rainfall. The climate of Australia and the Maritime Continent are affected by the cool (warm) SST anomalies over the southeast Indian Ocean region during the positive (negative) phase of the IOD^5^. The anomalously cool (warm) waters around Australia and the Maritime Continent during the positive (negative) phase of IOD reduce (enhance) rainfall over those countries. The IOD also has remote effect on the climate of Japan through modification of the Pacific-Japan teleconnection and it is also known to affect the summers of Europe due to the atmospheric teleconnections as a response to the IOD^6^. The IOD has large socio-economic impacts on many countries and hence predicting the IOD well in advance would benefit the affected societies. Various forecasting centers try to predict IOD using the coupled climate models at seasonal time scales. Such dynamical models are promising but are dependent on large computational as well as human resources. In this study we try to complement those efforts with a simpler model based on the machine learning technique of artificial neural networks (ANNs)^7^.

The ANNs are machine learning models which were initially developed to model the processes in neurons. The ANNs are nonlinear in nature and have been extensively used in the climate sciences for rainfall forecasts^8,9^, climate model parameterization^10^ and ENSO forecasting^11^. The ANNs based models essentially consist of an input layer, a number of hidden layers and an output layer. The number of neurons in the hidden layer is determined after a number of trials. In this study we forecast the IOD index for the IOD season for May-November from February-April conditions. The attributes of the ANN are derived from correlation analyses of sea surface temperature anomalies, 850 hPa and 200 hPa geopotential height anomalies. The geopotential height anomalies at mid- to high- latitudes well represent the atmospheric teleconnections to the equatorial SST anomalies. We could not find meaningful attributes to forecast the IOD index of May-November from December and January conditions confirming the “winter predictability barrier” of IOD discussed earlier using coupled dynamical models^12–15^. Hence the forecasts were started from February. The correlation analysis of the IOD index indicated that a single ANN model is not suitable for forecasting the IOD index for all the months from May to November. So, we developed ANN models for forecasting the IOD index for every month from May to November. The number of hidden layers is determined based on a number of trials. The number of neurons, in each layer which forecast IOD index with high correlation and low root mean square error, are chosen for each month. Similar exercises are carried out for forecasts initiated from February, March and April initial conditions. The number of hidden layers used for each model are shown in Table 1. An ensemble of forecasts is generated using 500 members to reduce the errors due to sampling and by varying the size of the training period we carried out ensemble forecasts to check the sensitivity of the ANN model forecasts to the length of the training period.

**Table 1:** Number of neurons used in the hidden layer for forecasting the IOD index of May-November from February, March and April initial conditions. The numbers such as 3:1 indicate two hidden layers with 3 and 1 neurons.

	May	June	July	August	September	October	November
February initial conditions	4:2	5	2	2	3	6	9
March initial conditions	3:2	2	8	5	5	2	12
April Initial conditions	3:1	2	6	3	8	4	3

The results of ANN forecasts are compared with persistence forecasts and also IOD index forecasts derived from the ensemble mean SST anomalies of seven models within the North American Multi-Model Ensemble (NMME)^16^. NMME is an experimental multi-model seasonal forecasting system consisting of coupled models from the United States of America and Canada. The details of the models and the model forecasts are well documented elsewhere^16^. The ANN and NMME model results are compared to persistence forecasts to check if the models have skill higher than just persistence of the IOD index of February-April to May-November.

## Results

### IOD index forecast from February

The ANN forecasts of May-November IOD Index from February were generated using the attributes identified by correlation analysis as described in the “Methods” section. The regions of SST anomalies used for the forecasts (Figs. S1–S3) are tabulated in Table 2. Similarly, the regions of 850 hPa geopotential height anomalies (Figs. S4, S5) and the 200 hPa geopotential height anomalies (Figs. S6 and S7) are tabulated in Tables 3 and 4 respectively. We used ANN models with varying input and hidden layers to forecast the IOD index of each month within the IOD season May-November, as the regions of significant correlation vary from month to month (Figs. S1–S7). The number of hidden layers used in the ANN models for the forecasts are given in Table 1.

**Table 2:** The regions of SST anomalies used as attributes to the ANN models.

	May	June	July	Aug	Sep	Oct	Nov
Feb	**(i)** 20°S–10°S; 70°E–110°E **(ii)** 50°S–40°S; 100°E–125°E	**(i)** 50°S–5°S; 160°E–190°E **(ii)** 40°S–30°S; 325°E–350°E **(iii)** 30°S–15°S; 80°E–110°E	**(i)** 35°S–25°S; 45°E–75°E **(ii)** 25°S–15°S; 65°E–90°E **(iii)** 50°S–5°S; 160°E–190°E **(iv)** 70°S–30°S; 320°E–340°E	**(i)** 20°S–10°S; 65°E–90°E **(ii)** 50°S–10°S; 160°E–190°E	**(i)** 20°S–10°S; 65°E–90°E **(ii)** 55°S–35°S; 60°E–80°E **(iii)** 30°S–20°S; 190°E–220°E **(iv)** 40°S–25°S; 245°E–270°E **(v)** 55°S–40°S; 245°E–285°E (**vi)** 55°S–40°S; 320°E–340°E **(vii)** 60°S–50°S; 100°E–130E **(viii)** 25°S–10°S; 325°E–350°E	**(i)** 20°S–10°S; 65°E–90°E **(ii)** 55°S–35°S; 60°E–80°E **(iii)** 30°S–20°S; 190°E–220°E **(iv)** 40°S–25°S; 245°E–270°E **(v)** 45°S–35°S; 320°E–340°E **(vi)** 60°S–50°S; 100°E–130E **(vii)** 25°S–10°S; 320°E–350°E	**(i)** 20°S–10°S; 65°E–90°E **(ii)** 35°S–25°S; 190°E–270°E **(iii)** 60°S–50°S; 100°E–115°E
Mar	**(i)** 38°S–25°S; 60°E–80°E **(ii)** 30°S–5S; 90°E–110°E **(iii)** 60°S–45°S; 100°E–135°E	**(i)** 40°S–30°S; 55°E–80°E **(ii)** 60°S–45°S; 110°E–145°E	**(i)** 40°S–25°S; 45°E–75°E **(ii)** 60°S–50°S; 100°E–140°E **(iii)** 50°S–25°S; 160°E–190°E	**(i)** 40°S–30°S; 50°E–80°E **(ii)** 15°S–5°S; 110°E–130°E **(iii)** 60°S–50°S; 100°E–130°E **(iv)** 20°N–30°N; 260°E–290°E	**(i)** 40°S–30°S; 50°E–80°E **(ii)** 20°S–0°N; 65°E––100°E **(iii)** 60°S–50°S; 100°E–130°E **(iv)** 20°N–30°N; 260°E–290°E **(v)** 45°S–35°S; 320°E–345°E **(vi)** 55°S–40°S; 245°E–285°E	**(i)** 40°S–30°S; 50°E–80°E **(ii)** 20°S–5°S; 65°E–110°E **(iii)** 60°S–50°S; 100°E–130°E (**iv)** 20°N–30°N; 260°E–290°E (**v)** 50°S–35°S; 320°E–345°E (**vi)** 55°S–40°S; 245°E–285°E	(**i)** 40°S–30°S; 50°E–80°E (**ii)** 20°S–5°S; 90°E–130°E (**iii)** 60°S–50°S; 100°E–130°E (**iv)** 20°N–30°N; 260°E–290°E (**v)** 50°S–35°S; 320°E–345°E (**vi)** 35°S–20°S; 190°E–225°E (**vii)** 35°S–25°S; 250°E–270°E
Apr	(**i)** 38°S; 20°S; 60°E–80°E (**ii)** 20°S–5°S; 90°E–105°E (**iii)** 55°S–40°S; 110°E–140°E (**iv)** 35°S–25°S; 340°E–360°E	(**i)** 40°S–25°S; 60°E–80°E (**ii)** 45°S–35°S; 210°E–230°E (**iii)** 55°S–40°S; 120°E–150°E	(**i)** 40°S–20°S; 45°E–75°E (**ii)** 0°N–15°N; 80°E–100°E (**iii)** 30°S–0°N; 110°E–160°E	(**i)** 35°S–20°S; 50°E–70°E (**ii)** 15°S–5°S; 110°E–130°E (**iii)** 20°N–30°N; 260°E–290°E	(**i)** 20°S–5°S; 130°E–160°E (**ii)** 20°N–30°N; 260°E–290°E (**iii)** 5°N–20°N; 160°E–200°E (**iv)** 55°S–40°S; 240°E–260°E	(**i)** 30°S–5°S; 110°E–180°E (**ii)** 20°N–30°N; 260°E–290E (**iii)** 5°N–20°N; 160°E–200°E (**iv)** 55°S–40°S; 240°E–280°E (**v)** 55°S–45°S; 100°E–130°E (**vi)** 60°S–50°S; 180°E–200°E	(**i)** 20°S–5°S; 90°E–110°E (**ii)** 20°N–30°N; 260°E–290°E (**iii)** 5°N–20°N; 160°E–200°E (**iv)** 60°S–50°S; 180°E–210°E (**v)** 40°N–50°N; 170°E–200°E

**Table 3:** The regions of 850 hPa geopotential height anomalies used as attributes to the ANN models.

	May	June	July	Aug	Sep	Oct	Nov
Feb	(**i)** 25°N–40°N; 150°E–170°E (**ii)** 45°N–55°N; 65°E–85°E	(**i)** 35°S–20°S; 70°E–90°E (**ii)** 35°S–30°S; 150°E–160°E (**iii)** 50°S–35°S; 325°E–345°E (**iv)** 50°N–65°N; 70°E–80°E (**v)** 25°N–35°N; 130°E–165°E	(**i)** 45°S–35°S; 325°E–340°E (**ii)** 25°N–35°N; 130°E–160°E	(**i)** 45°S–35°S; 325°E–340°E	(**i)** 25°N–40°N; 220°E–230°E (**ii)** 20°N–40°N; 270°E–300°E (**iii)** 50°N–60°N; 320°E–340°E	(**i)** 25°N–40°N; 220°E–240°E (**ii)** 20°N–40°N; 270°E–320°E (**iii)** 50°N–60°N; 315°E–330°E (**iv)** 60°N–70°N; 190°E–220°E (**v)** 40°S–20°S; 100°E–120°E	(**i)** 0°N–20°N; 120°E–180°E (**ii)** 55°N–65°N; 320°E–340°E (**iii)** 40°S–20°S; 100°E–120°E (**iv)** 45°S–35°S; 330°E–350°E
Mar	—	—	(**i)** 60°S–50°S; 40°E–70°E (**ii)** 50°N–70°N; 220°E–250°E (**iii)** 60°N–70°N; 110°E–140°E (**iv)** 20°S–0°N; 280°E–300°E	(**i)** 60°S–50°S; 40°E–65°E (**ii)** 45°N–60°N; 220°E–240°E	(**i)** 45°N–55°N; 170°E–200°E (**ii)** 10°N–20°N; 140°E–220°E (**iii)** 65°S–55°S; 200°E–240°E	(**i)** 45°N–55°N; 170°E–200°E (**ii)** 10°N–20°N; 140°E–220°E (**iii)** 50°N–70°N; 50°E–70°E (**iv)** 60°N–70°N; 330°E–350°E	(**i)** 0°N–20°N; 140°E–210°E (**ii)** 20°N–30°N; 260°E–280°E
Apr	(**i)** 35°S; 15°S; 245°E–265°E (**ii)** 20°N–35°N; 170°E–210°E (**iii)** 30°S–20°S; 80°E–110°E (**iv)** 15°N–30°N; 30°E–50°E (**v)** 0°N–20°N; 300°E–340°E (**vi)** 60°S–50°S; 150°E–170°E (**vii)** 30°N–40°N; 120°E–130°E	(**i)** 60°N–70°N; 170°E–200°E (**ii)** 35°S–15°S; 245°E–265°E	—	(**i)** 70°N; 80°N; 0°E–30°E	(**i)** 70°N–80°N; 0°E–30°E (**ii)** 10°N–30°N; 150°E–180°E	(**i)** 65°S–50°S; 190°E–220°E (**ii)** 50°N–60°N; 90°E–120°E (**iii)** 70°N–80°N; 330°E–30°E (**iv)** 10°N–30°N; 150°E–180°E	(**i)** 10°N–30°N; 140°E–200°E (**ii)** 65°S–50°S; 200°E–230°E (**iii)** 50°N–60°N; 90°E–120°E (**iv)** 45°S–30°S; 170°E–200°E (**v)** 60°N–80°N; 330°E–30°E

**Table 4:** The regions of 200 hPa geopotential height anomalies used as attributes to the ANN models.

	May	June	July	Aug	Sep	Oct	Nov
Feb	(**i)** 30°S–20°S; 115°E–140°E (**ii)** 30°S–20°S; 250°E–280°E (**iii)** 40°S–25°S; 65°E–85°E	(**i)** 50°N–65°N; 5°E–25°E (**ii)** 35°S–20°S; 260°E–280°E	(**i)** 20°N–30°N; 5°E–20°E (**ii)** 40°N–50°N; 55°E–65°E	—	—	(**i)** 50°N–65°N; 250°E–320°E (**ii)** 25°S–10°S; 220°E–250°E (**iii)** 35°S–20°S; 300°E–310°E	(**i)** 50°N–75°N; 230°E–320°E (**ii)** 35°N–45°N; 205°E–235°E (**iii)** 30°N–35°N; 270°E–300°E (**iv)** 60°S–45°S; 240°E–265°E (**v)** 10°S–10°N; 40°E–70°E (**vi)** 20°S–10°S; 200°E–240°E
Mar	—	(**i)** 30°N–40°N; 80°E–100°E	(**i)** 30°S–0°N; 320°E–350°E (**ii)** 10°N–25°N; 330°E–360°E (**iii)** 70°S–60°S; 250°E–280°E	(**i)** 30°N–50°N; 245°E–290°E (**ii)** 60°N–75°N; 210°E–240°E (**iii)** 40°N–55°N; 330°E–15°E (**iv)** 20°S–0°N; 320°E–350°E (**v)** 10°N–30°N; 340°E–360°E	(**i)** 15°S–5°S; 70°E–110°E (**ii)** 20°S–30°N; 290°E–350°E (**iii)** 15°S–5°S; 240°E–280°E (**iv)** 60°N–70°N; 310°E–340°E (**v)** 70°N–80°N; 210°E–270°E (**vi)** 65°S–50°S; 10°E–60°E	(**i)** 30°N–45°N; 250°E–285°E (**ii)** 20°S–5°S; 230°E–280°E (**iii)** 20°S–10°S; 60°E–110°E (**iv)** 20°S–20°N; 320°E–350°E (**v)** 60°N–80°N; 200°E–260°E (**v)** 60°S–50°S; 20°E–50°E (**vi)** 70°S–55°S; 200°E–240°E	(**i)** 40°N–50°N; 170°E–200°E (**ii)** 20°S–5°S; 230°E–280°E (**iii)** 20°S–10°S; 60°E–110°E (**iv)** 20°S–0°N; 320°E–350°E (**v)** 60°N–80°N; 200°E–260°E (**vi)** 40°N–50°N; 90°E–115°E (**vii)** 10°N–20°N; 340°E–10°E (**viii)** 10°N–20°N; 80°E–110°E
Apr	—	(**i)** 35°N–45°N; 185°E–205°E (**ii)** 40°S–30°S; 240°E–260°E (**iii)** 40°S–30°S; 315°E–330°E (**iv)** 30°N–50°N; 220°E–250°E	(**i)** 15°N–25°N; 10°E–20°E (**ii)** 30°S–15°S; 140°E–155°E (**iii)** 30°S–15°S; 305°E–315°E (**iv)** 40°N–50°N; 230°E–240°E	(**i)** 50°N–70°N; 70°E–100°E (**ii)** 30°S–10°S; 300°E–330°E (**iii)** 25°S–10°S; 130°E–150°E (**iv)**10°N–20°N; 5°E–30°E	(**i)** 15°S–5°S; 70°E–110°E (**ii)** 10°S–20°N; 110°E–170°E (**iii)** 10°S–20°N; 310°E–350°E (**iv)** 10°S–10°N; 220°E–260°E (**v)** 10°N–30°N; 70°E–110°E (**vi)** 40°S–30°S; 280°E–290°E (**vi)** 65°S–55°S; 270°E–280°E	(**i)** 20°S–20°N; 75°E–140°E (**ii)** 20°S–20°N; 310°E–340E (**iii)**50°S–30°S; 160°E–190E (**iv)** 70°S–50°S; 260°E–290E	(**i)** 10°S–10°N; 300°E–350°E (**ii**) 10°S–10°N; 10°E–170°E (**iii)** 55°S–55°S; 330°E–350°E (**iv)** 40°N–60°N; 330°E–10°E

**Figure 1:**
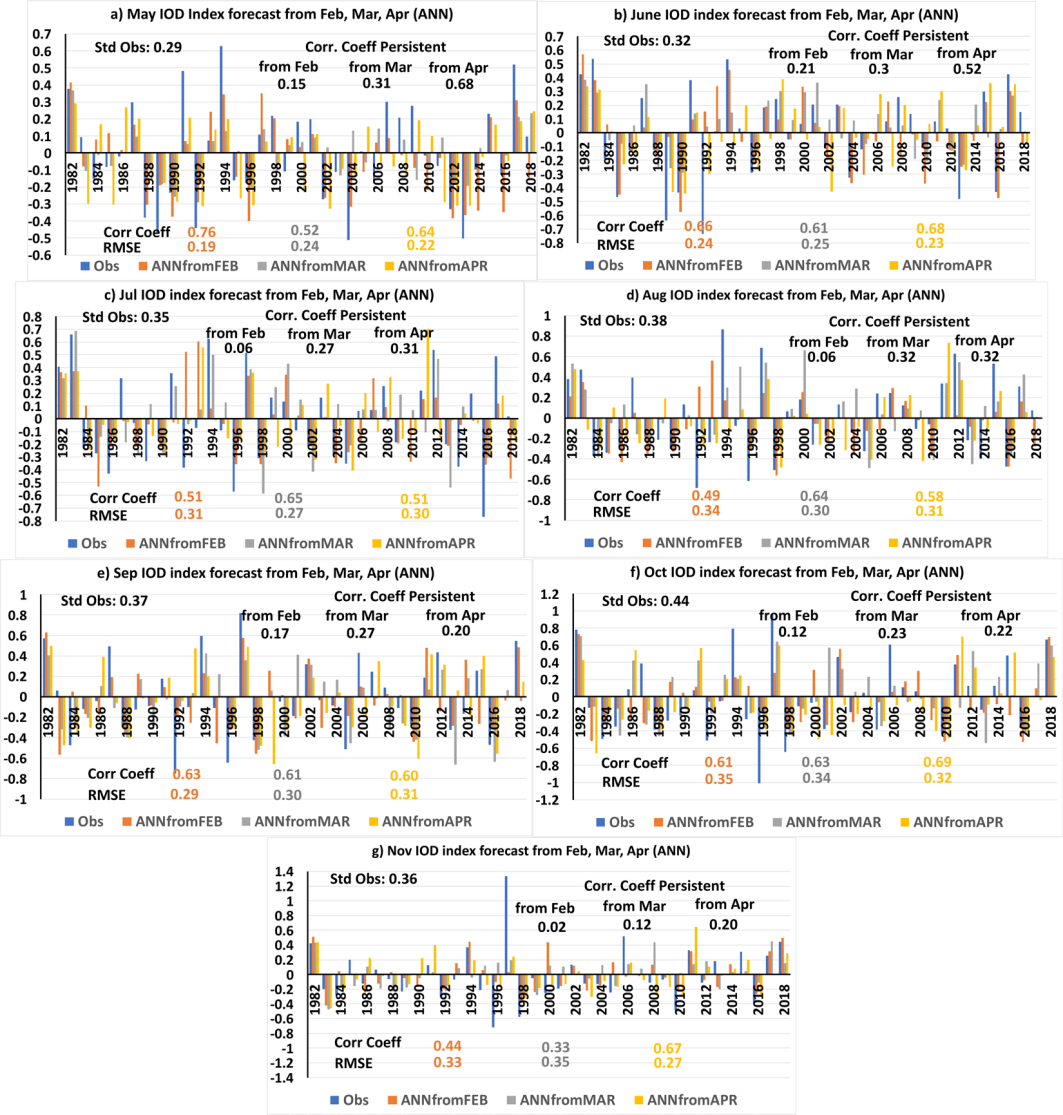
(**a–g**) Ensemble mean of 500 members forecast of IOD index for the months of May to November from February-April initial conditions using ANN models for the period 1982 to 2018. The panels were prepared using Microsoft EXCEL 2016 and merged with ImageMagick software (version 6.7.2-7) (https://imagemagick.org/).

The ensemble average of the 500-member IOD forecast for the months May to November initiated in February, March and April using the leave-one-out (or jackknife, see Methods) approach is analyzed (Fig. S8). Truncated forecasts highlighting the recent period from 1982–2018 are shown in Fig. 1 for comparison with the NMME forecasts.

**Figure 2:**
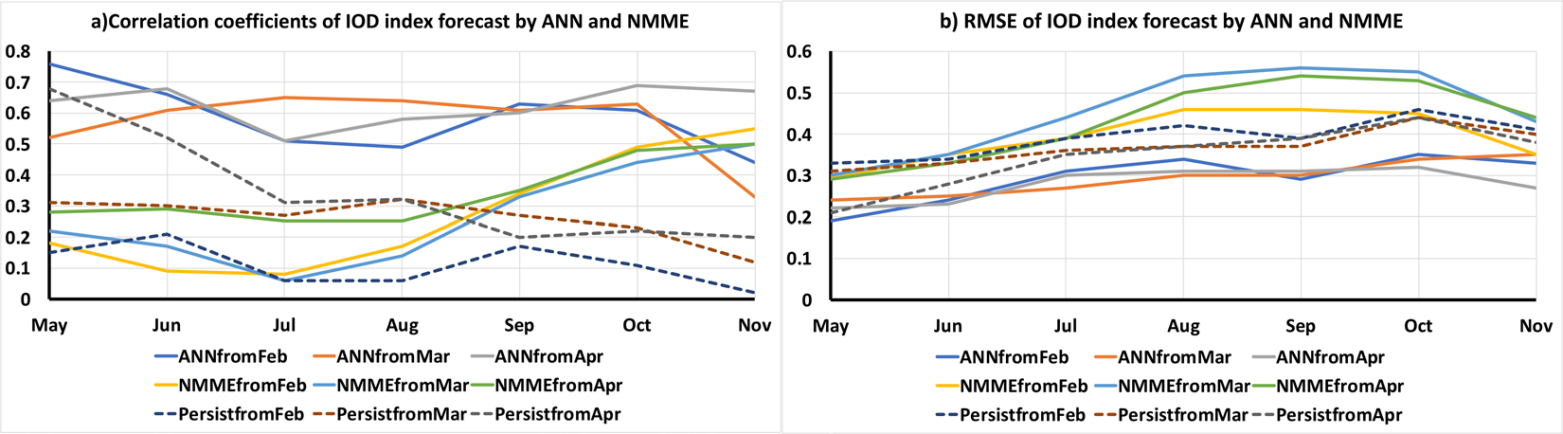
(**a**) ACC and (**b**) RMSE of the ANN, NMME and persistence forecasts for the months of May-November from February-April initial conditions. The panels were prepared using Microsoft EXCEL 2016 and merged with ImageMagick software (version 6.7.2-7) (https://imagemagick.org/).

To obtain an estimate of the skill of the models, the IOD forecasts by the ANN models (Fig. 1) are correlated with observed IOD indices and the Anomaly Correlation Coefficient (ACC) is calculated. The RMSE of the forecasts was also calculated to get an estimate of the deviation of the model forecasts from observed IOD index. The ACC and RMSE of the ANN forecast IOD index from February with the observed IOD indices for the period May-November are shown in Fig. 2. As seen from Fig. 2a, the ACC values of the February forecast of the ANN models varies from 0.44 to 0.76 with high (low) correlation in May (November) and the RMSE value varies between 0.19 to 0.35 over the May-November period. The ACC (RMSE) values of the persistence forecast, i.e. considering the observed IOD index of February to persist for the whole IOD season May-November are also shown in Fig. 2a,b. Comparing the ANN forecasts with the persistence forecasts (Fig. 2a,b) it is evident that the ANN forecasts have higher skill with higher ACC and lower RMSE values compared to the persistence forecasts. The result even holds if we consider the entire time period from 1949–2018. Though the ACC values of the ANN forecasts (Fig. S8) are lower for the forecasts for the entire period (1949–2018) compared to the values in the recent period from 1982–2018 (Fig. 1), the ACC values are higher than the ACC of the persistence forecast. The ACC values of ANN forecasts for the months May-November for the entire period 1949–2018 are 0.59, 0.57, 0.47, 0.47, 0.38, 0.38 and 0.29 respectively. Whereas the ACC values of the persistence forecast for the same period for May-November are 0.27, 0.20, 0.09, 0.14, 0.13, 0.12 and 0.11 respectively.

**Figure 3:**
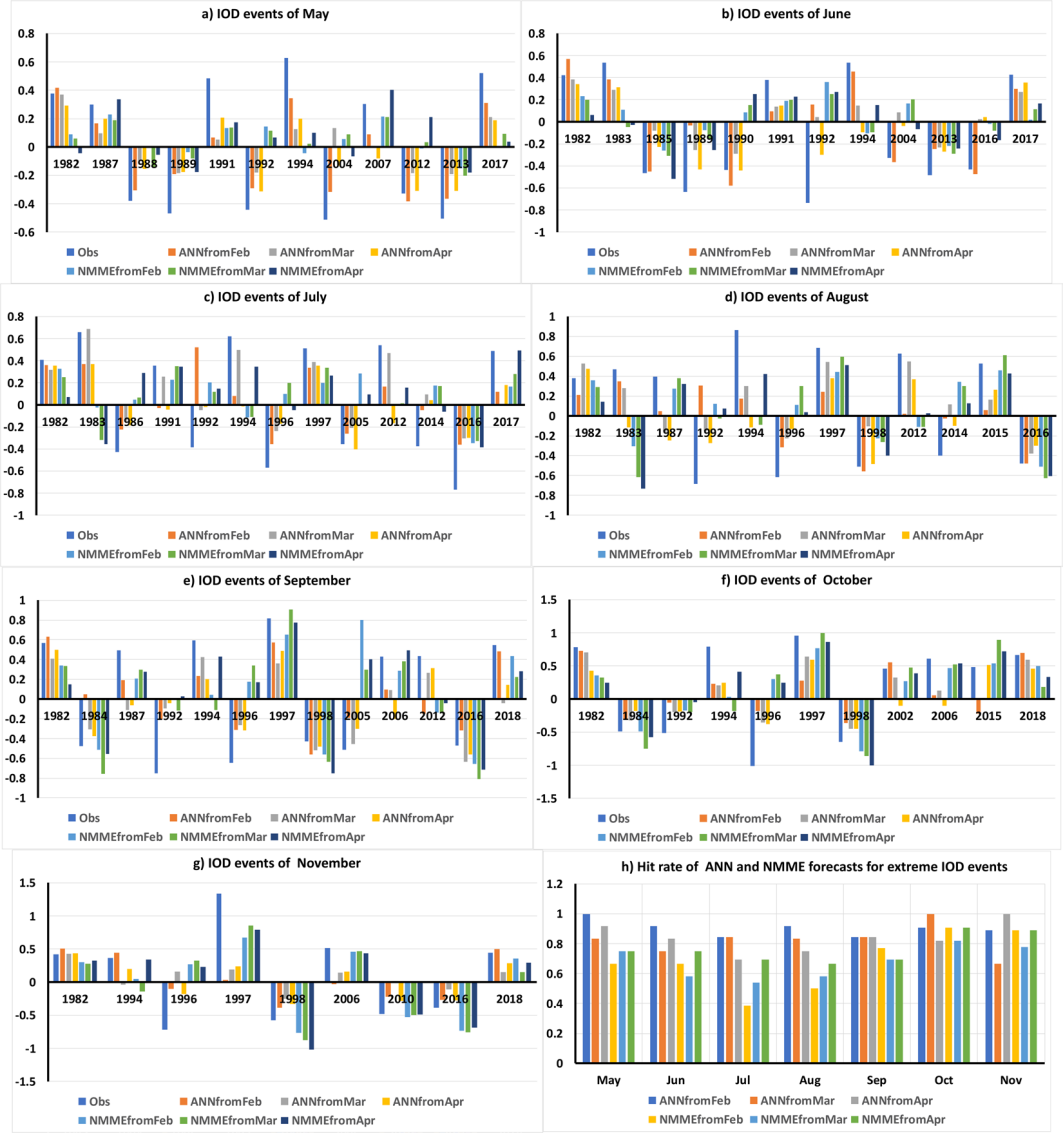
(**a–g**) IOD forecasts of extreme IOD events by ANN and NMME models for the months May-November from February-April initial conditions. (**h**) Hit rate of the ANN and the NMME models in forecasting extreme IOD events. The panels were prepared using Microsoft EXCEL 2016 and merged with ImageMagick software (version 6.7.2-7) (https://imagemagick.org/).

We analyzed the quality of the ANN models in forecasting the phase of the extreme positive and negative IOD events by identifying extreme events within the period 1982–2018. Extreme events are defined as events whose absolute magnitudes equal or exceed one standard deviation. In May, 12 extreme IOD events (6 positive and 6 negative IOD events) were observed during the period 1982–2018 (Fig. 3a). In June, five extreme positive IOD years with absolute amplitudes larger than or equal to one standard deviation (0.32 °C) and seven extreme negative IOD years were observed (Fig. 3b). From Fig. 3a it is evident that the ANN model forecasts from February could capture the phase of the strong positive and negative IOD events of May though the forecasts underestimate the amplitude of the events. The ANN models could forecast the phase of 11 of the 12 observed events correctly in the month of June (Fig. 3b). The hit rate score of the ANN models to forecast the May and June extreme IOD events from February is 1 and 0.91 respectively (Fig. 3h). The hit rate score is defined as the ratio of successful forecast of events to the total observed events. In July, 7 positive and 6 extreme negative IOD events with absolute amplitudes larger than or equal to 0.35 °C (one standard deviation) were observed (Fig. 3c). The ANN could forecast the phase of most the extreme IOD events realistically but failed to forecast the correct phase in years 1991 and 1992 (Fig. 3c). Similarly, in August of the 7 positive and 6 extreme negative IOD events with absolute amplitudes larger than or equal to 0.38 °C the ANN models could correctly forecast the phase of the all the IOD events except one in the year 1992 (Fig. 3d). The hit rate scores of the July and August forecasts from February are 0.84 and 0.91 (Fig. 3h) respectively. In September extreme positive IOD events (absolute amplitude greater than or equal to 0.37 °C) were observed in years 1982, 1987, 1994, 1997, 2006, 2012, and 2018 and negative IOD events in years 1984, 1992, 1996, 1998, 2005 and 2016 (Fig. 3e). The ANN models forecast the correct phase of IOD in all the years except in the years 1984 and 2012 (Fig. 3e). In October (November) 11 (5) positive and 4 (4) negative extreme strong IOD events with absolute amplitudes larger than or equal to 0.44 °C (0.36 °C) were observed. The ANN models could forecast most of the events realistically in October (November) except in the year 2015 (2006) Fig. 3f,g. The hit rate score of the September-November forecasts are 0.84, 0.90 and 0.88 (Fig. 3h).

The ANN model forecasts using 70% (80%) of the sample for training and 30% (20%) of the data for testing were also carried out for the entire period 1949–2018, as described in the “Methods” section, to test the robustness of the results obtained using the jackknife or Leave-One-Out method. Analyzing the forecasts, we find that the ensemble average of the 500 members of IOD index forecasts of May-November from February initial conditions, for the 1949–2018 period, using the 70% (80%) of the sample for training and 30% (20%) of the data for testing (Figs. S9 and S10) are not very different from the results obtained using jackknife approach (Fig. S8). The correlation coefficients and RMSE values of the forecasts are not very different from the ones obtained using jackknife method thereby indicating the forecasts of IOD index of the ANN models using jackknife approach to be robust.

**Figure 4:**
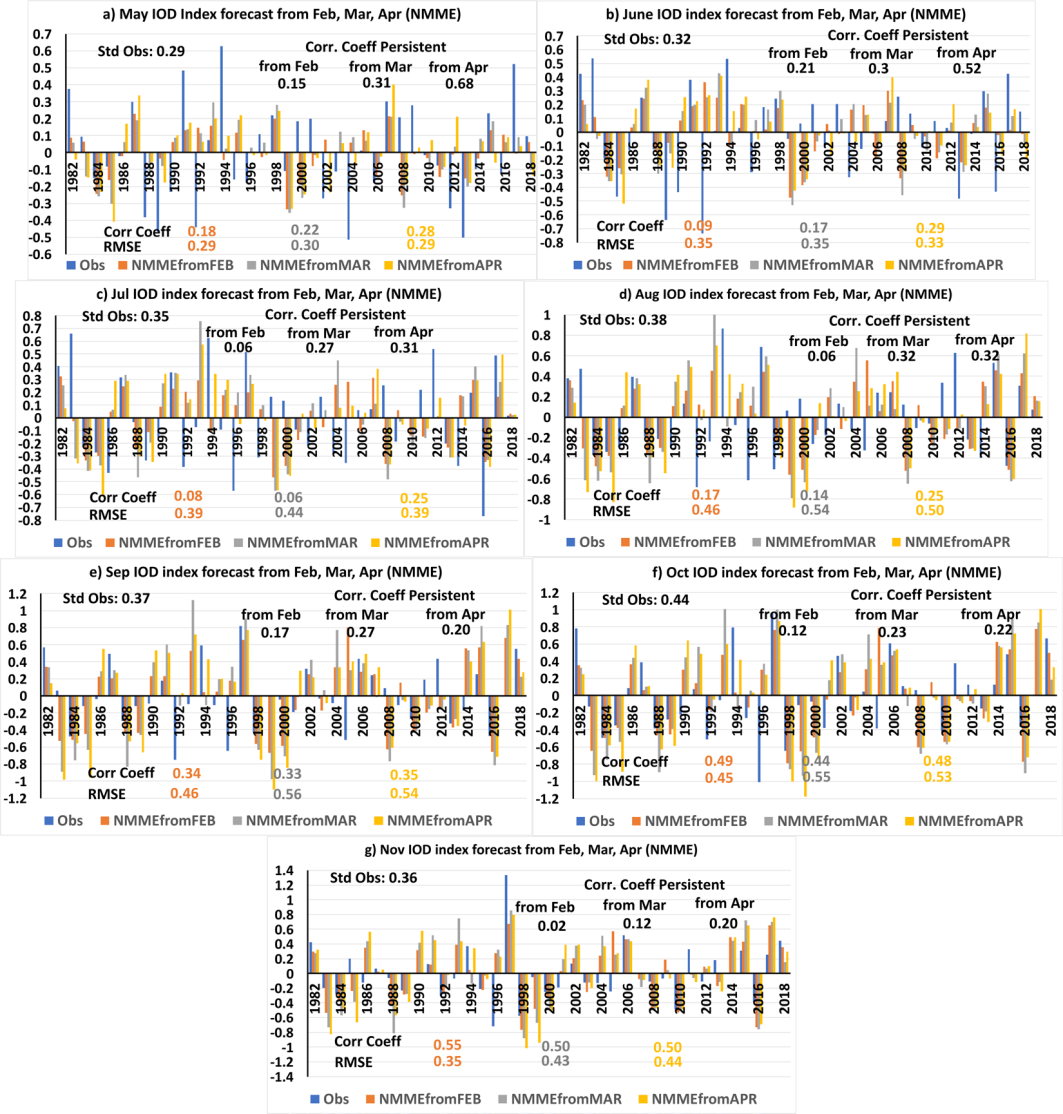
(**a–g**) The IOD index of May to November forecast by NMME models from February-April initial conditions. The panels were prepared using Microsoft EXCEL 2016 and merged with ImageMagick software (version 6.7.2-7) (https://imagemagick.org/).

Next, we compared the IOD forecasts from the ANN models with the ensemble average IOD forecasts from the state-of-the-art NMME numerical models. The IOD forecasts for May-November of the NMME models from the February, March and April initial conditions are shown in Fig. 4. The ACC and RMSE values of the NMME forecast from February initial conditions are shown in Fig. 2. Comparing the ACC values of the ensemble average of NMME model forecasts of IOD index from February initial conditions with persistence forecasts of February, we find that the ensemble mean of the NMME model is better than persistence forecasts in May and July-November (Fig. 2a). However, the NMME forecasts have higher RMSE values compared to persistence in August, September and October (Fig. 2b). Similarly, comparing the forecasts of IOD of the ANN and NMME models, we find the ANN models to perform better with higher ACC and lower RMSE values for the period May-October (Fig. 2). The November forecast of IOD of the NMME models has higher ACC value compared to the ANN forecast (Fig. 2a). However, the NMME models have higher RMSE values compared to the RMSE value of the ANN forecasts for November (Fig. 2b).

The NMME models have a lower hit rate score in forecasting the extreme IOD events for the period May-September and comparable score for October-November period relative to the hit rate score of ANN forecasts of extreme IOD events (Fig. 3h). In May the NMME models could successfully forecast the extreme IOD events for 8 events of the 12 observed events (Fig. 3a) with a hit score of 0.66 (Fig. 3h). In the period June-November the NMME model forecasts have a hit score of 0.66, 0.38, 0.5, 0.77, 0.91, 0.89 respectively (Fig. 3h).

### IOD index forecast from March

The attributes to the ANN models for the IOD forecasts from March are derived from the identified significant regions of SST and geopotential height anomalies at 850 hPa and 200 hPa levels (Figs. S11–S17; Tables 2–4). As is evident from Tables 2–4, these regions vary each month for the period May-November. Hence, similar to the forecasts from February, we used ANN models with varying input and hidden layers (Table 1) to forecast the IOD index from March.

The IOD forecasts of the ANN models from March is shown in Fig. 1 for the period 1982–2018. The ACC (RMSE) values of the ANN forecast IOD indices for May-November forecasts from March conditions are shown in Fig. 2. Comparing the ANN forecasts from March with persistence forecasts we find the ACC values of the ANN forecast IOD index are higher than that of persistence forecasts (Fig. 2a). Also, the RMSE values of the ANN forecasts are lower than the RMSE values of the persistence forecast (Fig. 2b). Similar to February forecasts, the March forecasts also have lower ACC values of the ANN forecasts when the entire period (1949–2018) is considered (Fig. S8) when compared with the values of the recent period (1982–2018) (Fig. 1). The ACC values of ANN forecasts for the months May-November considering the entire period 1949–2018 are 0.34, 0.46, 0.62, 0.47, 0.56, 0.54 and 0.34 respectively. However, the ANN forecasts for the entire period have higher ACC values compared to the persistence forecasts which have ACC values 0.31, 0.29, 0.18, 0.25, 0.19, 0.23, 0.25 for the May-November period.

Analyzing the ANN model forecasts from March conditions we find that the ANN models could successfully forecast the phase of all the extreme positive IOD events in May (Fig. 3a) and June (Fig. 3b) though the amplitude is underestimated. The ANN models could forecast the correct phase of all the strong negative IOD events of May except in the year 2004 (Fig. 3a). In June the models failed to forecast the correct phase of extreme negative IOD of years 1992, 2004 and 2016 (Fig. 3b). In July the ANN models failed to forecast the correct phase of IOD in years 2014 and 2017 (Fig. 3c). Similarly, in August the ANN models from March failed to correctly forecast the phase in 1987 and 2014 (Fig. 3d). The ANN models could also forecast most of the extreme IOD events of September from March conditions for most of the years except in years 1987 and 2018 (Fig. 3e). The extreme IOD events of October were successfully forecast by the ANN model from March conditions for all the events (Fig. 3f). In November, the ANN models could forecast the strong positive IOD events of years 1982, 1997, 2006 and 2018 and strong negative IOD events of years 1998 and 2016 (Fig. 3g) but failed to forecast the correct phase of the IOD events in 1994, 1996 and 2010 (Fig. 3g). The hit rates of the ANN forecasts from March conditions are shown in Fig. 3h.

On comparing the ACC and RMSE of the IOD forecasts using jackknife method (Fig. S8) with the forecasts using only 70% and 80% of the sample data for training (Figs. S9 and S10) we find the ACC and RMSE values to be comparable. This indicates the IOD forecasts using the jackknife method to be robust.

The IOD indices forecast by NMME models from the March initial conditions are shown in Fig. 4. The ACC and RMSE values of the NMME forecast IOD indices from March initial conditions for the months May-November are shown in Fig. 2. The March persistence forecast has higher ACC compared to the NMME forecasts for the months May to August (Fig. 2). From September to November the NMME models have higher ACC values compared to persistent forecasts (Fig. 2a). As seen from Fig. 2, the ANN forecasts from March have higher ACC and low RMSE values compared to the NMME forecasts for the period May-October (Fig. 2). In November the NMME forecast has higher ACC value but also high RMSE values (Fig. 2) compared to the ANN forecast.

Comparing the forecasts of extreme IOD events and their hit rate scores between the NMME forecasts and ANN forecasts (Fig. 3), it is evident that the ANN models have better skill in forecasting the correct phase of the extreme IOD events for the entire period May-November from March (Fig. 3).

### IOD index forecast from April

The significant regions from which the SST anomalies, 850 hPa and 200 hPa geopotential height anomalies for the April forecasts are derived (Figs. S18–S24) are given in Tables 2–4. ANN models using the data from the identified regions were used to forecast the IOD index for the period May-November.

Figure 1 shows the ANN forecast of IOD index from April conditions for the period 1982–2018. The ACC values of the IOD forecasts from April for the months Mar-November are shown in Fig. 2a. Similar to the forecasts from February and March the ACC values are higher than April persistence forecasts (Fig. 2a). The RMSE of the ANN forecasts are lower than the RMSE values of the persistent forecasts (Fig. 2b). Similar to the February and March ANN forecasts, the ACC values of the ANN forecasts from April conditions for the period 1949–2018 (Fig. S8) are less than the values for the period 1982–2018. The ACC values of ANN forecasts for the months June-Nov considering the entire period 1949–2018 are 0.52, 0.54, 0.44, 0.53, 0.55 and 0.48 respectively. However, the ANN forecasts for the June-November period have higher ACC values compared to the persistent forecasts of 0.43, 0.16, 0.11, −0.03, 0.05, 0.18 for June-November period considering the period 1949–2018. For the month of May, the persistence has slightly higher ACC (0.61) compared to the ANN forecast (0.59) (Fig. S8).

The extreme IOD events in May were forecast successfully by the ANN models from April conditions except for the year 2007 (Fig. 3a). In June, the ANN forecasts of the extreme IOD events failed for years 1994 and 2016 (Fig. 3b). The ANN models from April failed to forecast the correct phase of extreme IOD events in years 1991, 1994, 2012 and 2014 in July (Fig. 3c). In August extreme IOD events were successfully forecast by the ANN models for most of the years except in years 1983, 1987 and 1994 (Fig. 3d). In September, October and November the ANN models could successfully forecast the phase of all the extreme negative IOD events (Fig. 3e–g). However, the ANN models had difficulty in in forecasting the phase of some extreme positive IOD events of years 1987 and 2006 of September events (Fig. 3e) and years 2002 and 2006 of the October events (Fig. 3f). The hit ratio of the ANN forecast from April conditions are shown in Fig. 3h.

Similar to February and March forecasts, the April forecasts using jackknife approach are robust and the results are not very sensitive to the change in the length of the training period (Figs. S8–S10).

The IOD forecast of the NMME models from the April initial conditions is shown in Fig. 4 and the ACC, RMSE values are shown in Fig. 2. Comparing the ACC values of NMME forecasts with persistence forecast for the period May-November (Fig. 2), it is evident that the persistence forecasts are better than NMME forecasts of IOD index for the months May-August. From September to November the NMME models have higher ACC values compared to persistence forecasts (Fig. 2a). The ANN forecasts from April have higher ACC and low RMSE values compared to the NMME forecasts for the entire period May-November (Fig. 2).

Analyzing the quality of the NMME models in forecasting the extreme IOD events, we find that the April initialized NMME model has lower skill in forecasting the extreme IOD events compared to the ANN model forecasts for May-September and in November (Fig. 3).

## Discussion

In the present study we provided forecasts of the IOD index of May to November, the period covering the various phases of IOD, from February, March and April initial conditions. The results of the ANN forecasts were compared with the persistence forecasts and the ensemble mean forecast of the NMME models.

The predictors for the ANN models were identified by correlation analysis of the IOD index of May-November with the SST, 850 hPa and 200 hPa geopotential height anomalies of February, March and April. The area averaged anomalies over the regions of significant correlation are then used as attributes to the ANN models. Using these attributes along with the observed IOD index, the ANN models acquire non-linear relationships between the IOD index and the predictors in the training period. The weights of the non-linear relations obtained in the training period are then used to forecast the IOD index of the test period.

The correlation of the IOD index of various months with the February, March and April SST, 850 hPa and 200 hPa geopotential height anomalies (Figs. S1–S7 and S11–S24) reveals that the regions of significant correlations vary from month to month. Hence, separate ANN models are required to predict the monthly IOD index within the IOD season of May-November. Based on these results, we used ANN models with varying hidden layers (Table 1) and inputs (Tables 2–4) to predict the monthly IOD indices.

Analyzing the correlation of the May-November IOD indices with the February SST anomalies (Figs. S1–S3) all months have regions of significant negative correlation in the South Indian Ocean indicating the IOD of May-November to have signature in the February SST anomalies in the South Indian Ocean. In the month of May, the region of significant negative correlation is in the Southeast Indian Ocean nearer to Sumatra (Fig. S1a). In the other months of July-November, the region of significant negative correlation is seen over the Southcentral Indian Ocean (Fig. S1c–g). The regions of significant negative correlations in the South Indian Ocean expand northwards and are seen as the eastern pole of the IOD on correlating the May-November IOD indices with March (Figs. S7, S8 and S9a,d–g) and April (Figs. S18, S19 and S20a,c–g) SST anomalies. The above analysis indicates that the signatures of the IOD from May-November are evident in the February-April SST anomalies. Such an existence of IOD precursor in the region nearer to the dominant pole of the IOD helps the ANN models train better and hence those forecast IOD indices of May-November with higher skills. Apart from the region of negative correlation in the South Indian Ocean, a region of positive correlation is seen in the subtropical regions of South Indian Ocean. The region of positive correlation coefficient is significant in July, September and October in the correlation of IOD indices of July, September and October with February SST anomalies (Fig. S1c,e,f). The regions are also seen in the 1982–2018 period (Figs. S2 and S3). Analyzing the 850 hPa and 200 hPa geopotential height anomalies for both the periods 1949–2018 and 1982–2018 (Figs. S4–S7) we find, for most of the months, that the regions of positive correlations in the sub-tropical February SST anomalies are coincident with regions of positive correlation of geopotential height anomalies. However, the correlation coefficients of 850 hPa and 200 hPa geopotential height anomalies over those regions are higher in the 1982–2018 period compared to the 1949–2018 period. These results indicate that the SST anomalies over the sub-tropical South Indian Ocean are determined by the phase of quasi-stationary geopotential height anomalies. The same holds true for the regions of positive correlation in the sub-tropical South Indian Ocean in the correlation of IOD indices of May-November with March SST anomalies. Further, regressing the surface wind anomalies of April with the IOD indices of May-November (Figs. S25–S26) shows the surface wind anomalies from the tropical regions are directed towards the regions of positive correlation in the subtropical South Indian Ocean. The surface wind anomalies can help in propagating the SST anomalies from the tropical region to the sub-tropical region and maintain the SST anomalies over the sub-tropical South Indian regions. The region of positive correlation in the sub-tropical South Indian Ocean seen in the correlation of Feb SST anomalies with IOD index of May (Figs. S1–S3) becomes significant in the correlation of March SST anomalies with May IOD index (Figs. S11–S13). The region expands and extends towards Madagascar in the correlation of April SST anomalies with May IOD index (Figs. S18–S20) and is seen merging with the region of positive correlation coefficients in the west Indian Ocean in May (Figs. S18–20). The regions of significant correlation in the SST anomalies in February-April (Figs. S1–S3, S11–S13 and S17–19) in the mid- to high-latitudes in the Pacific and Atlantic Oceans have same sign as the correlation of 850 hPa and 200 hPa geopotential height anomalies (Figs. S4–S7, S14–S17 and S21–S24) thereby indicating the SST anomalies over those regions to be a response to a quasi-stationary geopotential height anomaly. To dynamically explain the contribution of these regions to the forecast of IOD index requires model sensitivity experiments which we plan do in a future study.

Using the identified predictors/input variables we predicted the IOD index for the IOD season from May to November. We generated an ensemble of ANN forecasts using 500 members using a jackknife or Leave-One-Out approach. We also verified the results by generating an ensemble of 500 members with 70% (80%) of the sample for training and forecasting for 30% (20%) remaining sample. The ensemble mean of the forecasts of the IOD index indicates that the ANN models are capable of forecasting the IOD index well in advance with excellent skills (particularly for 1982–2018 period) and far above the skills from the persistence forecasts. The ANN forecasts also outperform the forecasts of the NMME models. We analyzed the quality of the ANN and NMME models in forecasting the phase of the extreme positive and negative IOD events. The hit score of the ANN models in forecasting the extreme IOD events is higher compared to that of the NMME models for the target months (Fig. 3h).

The simultaneous correlation of the observed IOD index with the HadISST anomalies shows regions of significant correlation in the equatorial Pacific and in the Indian Ocean (Fig. S27) in all the months May-November. However, the simultaneous correlation of observed IOD index with the NMME model SST anomalies does not have significant correlations over the equatorial Pacific and in the Indian Ocean in the months May-August (Figs. S28–S30). Nevertheless, the NMME model capture well the regions of significant regions in the Pacific and in the South Indian Ocean from September-November (Figs. S28–S30), the period when the NMME models show high skill in the IOD forecast. The above analysis indicates that the NMME models have biases in forecasting the SST in the months May-August from all the initial conditions February-April which leads to poor skill in the forecast of IOD index. The ANN model forecasts are solely dependent on past observed data and hence have higher skills compared to the NMME models in forecasting the IOD index.

## Methods

### Identification of predictors (input) for ANNs

The attributes for the ANNs are identified using the Pearson’s correlation analysis of the monthly IOD index with the monthly sea surface temperature (SST) anomalies and geopotential height anomalies at 850 hPa and 200 hPa levels for the period spanning 1949 to 2018. We used the Hadley Center Sea Ice and Sea Surface Temperature data set (HadISST)^17^ for the SST. HadISST dataset is global in extent with a horizontal resolution of 1° × 1° and is available from Jan 1870 to present day. We chose the period covering Jan 1949 to Dec 2018 for our study to match the period of availability of the geopotential height fields from the National Centers for Environmental Prediction (NCEP)/National Center for Atmospheric Research (NCAR) reanalysis^18^. The SST and NCEP/NCAR reanalysis data from the satellite era i.e. after 1982 is considered reliable compared to the pre-satellite era data. We first carried out the correlation analysis at various lags for the period from 1982 to 2018 and checked if the regions are statistically significant for the data extended backwards to 1949. Only those regions which are statistically significant in both the periods i.e. 1982–2018 and 1949–2018, are considered as predictors for the ANNs. To make the identified regions robust, we also carried out the correlation analysis with the National Oceanic and Atmospheric Administration (NOAA) Optimum Interpolation (OI) sea surface temperature V2^19^ data set for the period of 1982 to 2018. The regions which are statistically significant among the SST datasets are chosen as the SST input to ANNs. Similarly, the geopotential height anomalies significant in both the periods 1982–2018 and 1949–2018 are considered as input to the ANNs. The geopotential height anomalies in the mid- to high-latitudes are barotropic in nature, hence if a region in mid- and high- latitudes is significant at both 850 hPa and 200 hPa levels, we considered the attributes only from the 850 hPa geopotential height anomalies. The regions of significant correlations between 80°S to 80°N are only considered in the study.

The anomalies are generated for both SST and geopotential height with 1982–2018 as the base period and the anomalies are linearly detrended. The IOD index used in the study is generated from the detrended HadISST anomalies for the period 1949–2019 as the gradient between the western equatorial Indian Ocean (50°E–70°E and 10°S–10°N) and the Southeastern equatorial Indian Ocean (90°E–110°E, 10°S–0°N)^1^. Similarly, the IOD index is generated from the OIV2 detrended anomalies for the correlation analysis with OIV2 dataset.

As the IOD starts in May-June and peaks in September-October and ends by November-December, we forecasted the IOD index for the IOD season, from May to November, starting from February, March and April initial conditions.

### Artificial neural networks (ANNs)

Artificial Neural Networks (ANNs) are tools used in machine learning which mimic the functioning of neurons in the human brain. Similar to the human brain, the ANN also learns from the past data and makes decisions for future. An ANN consist of input, output and hidden layers. ANNs have been used in many fields for classification and regression studies to model processes. We used the “neuralnet” library in the R software environment to apply the ANNs technique to forecast the IOD index. The methodology we followed to forecast the IOD index is:

(i). Identify the input parameters (attributes) using Pearson’s correlation analysis as described above
(ii). The input parameters are normalized using max-min normalization method. This technique gets all the scaled data in the range [0,1].
(iii). We used the jackknife^20^ approach of leave-one-out to get the weights for the training period (69 years) and applied the weights to the verification year (1 year) for all the years from 1949 to 2018. In the jackknife approach the data is divided into training (69 years) and testing period (1 year). This process is repeated to cover the entire period of 70 years. The ANNs forecasts are known to be sensitive to the training data sample. Hence, we used the bootstrap method to generate an ensemble of 500 forecasts by using 500 samples from the training period. For example, to forecast the IOD index of 2018, we generated 500 training data sets by sampling the years from 1949 to 2017 with replacement. Using the 500 training data samples we generated 500 forecasts for the year 2018. This process is repeated for all the years. This procedure ensures that the testing data is not included in the training data set while applying the bootstrapping method
(iv). To further cross-validate the forecasts for dependency of the forecasts on the length of the sample size, we divided the data of the 500 samples into 70% training and 30% testing data, and 80% training and 20% testing data with the year to be forecast included in the testing data and generated an ensemble of 500 forecasts. This procedure was repeated for all the years.

### North American multi-model ensemble (NMME)

The NMME forecast data is freely available from the International Research Institute (https://iridl.ldeo.columbia.edu/SOURCES/.Models/.NMME/). The NMME forecasts are from 1982 to present day at different lead times. We chose the forecasts of 7 models which have continuous data from 1982 to 2018 i.e. CMC1-CanCM3, CMC2-CanCM4, COLA-RSMAS-CCSM3, COLA-RSMAS-CCSM4, GFDL-CM2p1-aer04, GFDL-CM2p5-FLOR-A06, GFDL-CM2p5-FLOR-B01. The ensemble average of 11 members of the model forecast SSTs are used for analysis. The monthly SST anomalies are generated from the forecast SST fields by taking 1982–2018 as the base period. The SST anomalies are detrended and the IOD index is derived from the detrended anomalies of the ensemble mena of the seven models.

## Supplementary information

Supplementary Information.
