# Supplementary material for: A machine learning based prediction system for the Indian Ocean Dipole
Source: Sci Rep. 2020 Jan 14;10:284. doi: 10.1038/s41598-019-57162-8 (PMC6959259; doi:10.1038/s41598-019-57162-8)
Supplement: Supplementary file 1 — Supplementary Information. [file 41598_2019_57162_MOESM1_ESM.pdf]

A machine learning based prediction system for the Indian Ocean Dipole

J. V. Ratnam[1*], H.A. Dijkstra[2], Swadhin K. Behera[1]

[1]Application Laboratory, Japan Agency for Marine-Earth Science and Technology, Yokohama Japan

[2]Institute for Marine and Atmospheric research Utrecht, Utrecht university, Utrecht, The Netherlands

Supplementary information

The supplementary figures referred in the main article are presented in this document.

List of Supplementary figures

Fig S1.a-g) Spatial distribution of correlation coefficients of IOD index of May to November with HadISST February SST anomalies (°C) for the period 1949-2018. The correlations significant at 95% using Student's two tailed test are marked with crosses. The rectangular boxes represent the regions which are used as input to the ANN models.

The figure was prepared using The NCAR Command Language (version 6.4.0) [Software]. (2017). Boulder, Colorado: UCAR/NCAR/CISL/TDD. http://dx.doi.org/10.5065/D6WD3XH5

Fig S2.a-g) Spatial distribution of correlation coefficients of IOD index of May to November with HadISST February SST anomalies (°C) for the period 1982-2018. correlations significant at 95% using Student's two tailed test are marked with crosses. The rectangular boxes represent the regions which are used as input to the ANN models.

The figure was prepared using The NCAR Command Language (version 6.4.0) [Software]. (2017). Boulder, Colorado: UCAR/NCAR/CISL/TDD. http://dx.doi.org/10.5065/D6WD3XH5

Fig S3.a-g) Spatial distribution of correlation coefficients of IOD index of May to November with OIV2 February SST anomalies (°C) for the period 1982-2018. The correlations significant at 95% using Student's two tailed test are marked with crosses. The rectangular boxes represent the regions which are used as input to the ANN models.

The figure was prepared using The NCAR Command Language (version 6.4.0) [Software]. (2017). Boulder, Colorado: UCAR/NCAR/CISL/TDD. http://dx.doi.org/10.5065/D6WD3XH5

Fig S4.a-g) Spatial distribution of correlation coefficients of IOD index of May to November with 850hPa geopotential height anomalies (m) of February for the period 1949-2018. The correlations significant at 95% using Student's two tailed test are marked with crosses. The rectangular boxes represent the regions which are used as input to the ANN models.

The figure was prepared using The NCAR Command Language (version 6.4.0) [Software]. (2017). Boulder, Colorado: UCAR/NCAR/CISL/TDD. http://dx.doi.org/10.5065/D6WD3XH5

Fig S5.a-g) Spatial distribution of correlation coefficients of IOD index of May to November with 850hPa geopotential height anomalies (m) of February for the period 1982-2018. The correlations significant at 95% using Student's two tailed test are marked with crosses. The rectangular boxes represent the regions which are used as input to the ANN models.

The figure was prepared using The NCAR Command Language (version 6.4.0) [Software]. (2017). Boulder, Colorado: UCAR/NCAR/CISL/TDD. http://dx.doi.org/10.5065/D6WD3XH5

Fig S6.a-g) Spatial distribution of correlation coefficients of IOD index of May to November with 200hPa geopotential height anomalies (m) of February for the period 1949-2018. The correlations significant at 95% using Student's two tailed test are marked with crosses. The rectangular boxes represent the regions which are used as input to the ANN models.

The figure was prepared using The NCAR Command Language (version 6.4.0) [Software]. (2017). Boulder, Colorado: UCAR/NCAR/CISL/TDD. http://dx.doi.org/10.5065/D6WD3XH5

Fig S7.a-g) Spatial distribution of correlation coefficients of IOD index of May to November with 200hPa geopotential height anomalies (m) of February for the period 1982-2018. The

correlations significant at 95% using Student's two tailed test are marked with crosses. The rectangular boxes represent the regions which are used as input to the ANN models.

The figure was prepared using The NCAR Command Language (version 6.4.0) [Software]. (2017). Boulder, Colorado: UCAR/NCAR/CISL/TDD. http://dx.doi.org/10.5065/D6WD3XH5

Fig S8a-g. Ensemble mean of 500 members forecast of IOD index for the months of May to November from February, March and April initial conditions using ANN models for the period 1949 to 2018.

The panels were prepared using Microsoft EXCEL 2016 and merged with ImageMagick software (version 6.7.2-7) (https://imagemagick.org/)

Fig S9a-g. Ensemble mean of 500 members forecast of IOD index for the months of May to November from February, March and April initial conditions using ANN models for the period 1949 to 2018 using 70 % of each sample for training.

The panels were prepared using Microsoft EXCEL 2016 and merged with ImageMagick software (version 6.7.2-7) (https://imagemagick.org/)

Fig S10a-g. Ensemble mean of 500 members forecast of IOD index for the months of May to November from February, March and April initial conditions using ANN models for the period 1949 to 2018 using 80 % of each sample for training.

The panels were prepared using Microsoft EXCEL 2016 and merged with ImageMagick software (version 6.7.2-7) (https://imagemagick.org/)

Fig S11.a-g) Spatial distribution of correlation coefficients of IOD index of May to November with HadISST March SST anomalies (°C) for the period 1949-2018. The correlations significant at 95% using Student's two tailed test are marked with crosses. The rectangular boxes represent the regions which are used as input to the ANN models.

The figure was prepared using The NCAR Command Language (version 6.4.0) [Software]. (2017). Boulder, Colorado: UCAR/NCAR/CISL/TDD. http://dx.doi.org/10.5065/D6WD3XH5

Fig S12.a-g) Spatial distribution of correlation coefficients of IOD index of May to November with HadISST March SST anomalies (°C) for the period 1982-2018. The correlations significant at 95% using Student's two tailed test are marked with crosses. The rectangular boxes represent the regions which are used as input to the ANN models.

The figure was prepared using The NCAR Command Language (version 6.4.0) [Software]. (2017). Boulder, Colorado: UCAR/NCAR/CISL/TDD. http://dx.doi.org/10.5065/D6WD3XH5

Fig S13.a-g) Spatial distribution of correlation coefficients of IOD index of May to November with OIV2 March SST anomalies (°C) for the period 1982-2018. The correlations significant at 95% using Student's two tailed test are marked with crosses. The rectangular boxes represent the regions which are used as input to the ANN models.

The figure was prepared using The NCAR Command Language (version 6.4.0) [Software]. (2017). Boulder, Colorado: UCAR/NCAR/CISL/TDD. http://dx.doi.org/10.5065/D6WD3XH5

Fig S14.a-g) Spatial distribution of correlation coefficients of IOD index of May to November with 850hPa geopotential height anomalies (m) of March for the period 1949-2018. The correlations significant at 95% using Student's two tailed test are marked with crosses. The rectangular boxes represent the regions which are used as input to the ANN models.

The figure was prepared using The NCAR Command Language (version 6.4.0) [Software]. (2017). Boulder, Colorado: UCAR/NCAR/CISL/TDD. http://dx.doi.org/10.5065/D6WD3XH5

Fig S15.a-g) Spatial distribution of correlation coefficients of IOD index of May to November with 850hPa geopotential height anomalies (m) of March for the period 1982-2018. The correlations significant at 95% using Student's two tailed test are marked with crosses. The rectangular boxes represent the regions which are used as input to the ANN models.

The figure was prepared using The NCAR Command Language (version 6.4.0) [Software]. (2017). Boulder, Colorado: UCAR/NCAR/CISL/TDD. http://dx.doi.org/10.5065/D6WD3XH5

Fig S16.a-g) Spatial distribution of correlation coefficients of IOD index of May to November with 200hPa geopotential height anomalies (m) of March for the period 1949-2018. The correlations significant at 95% using Student's two tailed test are marked with crosses. The rectangular boxes represent the regions which are used as input to the ANN models.

The figure was prepared using The NCAR Command Language (version 6.4.0) [Software]. (2017). Boulder, Colorado: UCAR/NCAR/CISL/TDD. http://dx.doi.org/10.5065/D6WD3XH5

Fig S17.a-g) Spatial distribution of correlation coefficients of IOD index of May to November with 200hPa geopotential height anomalies (m) of March for the period 1982-2018. The correlations significant at 95% using Student's two tailed test are marked with crosses. The rectangular boxes represent the regions which are used as input to the ANN models.

The figure was prepared using The NCAR Command Language (version 6.4.0) [Software]. (2017). Boulder, Colorado: UCAR/NCAR/CISL/TDD. http://dx.doi.org/10.5065/D6WD3XH5

Fig S18.a-g) Spatial distribution of correlation coefficients of IOD index of May to November with HadISST April SST anomalies (ºC) for the period 1949-2018. The correlations significant at 95% using Student's two tailed test are marked with crosses. The rectangular boxes represent the regions which are used as input to the ANN models.

The figure was prepared using The NCAR Command Language (version 6.4.0) [Software]. (2017). Boulder, Colorado: UCAR/NCAR/CISL/TDD. http://dx.doi.org/10.5065/D6WD3XH5

Fig S19.a-g) Spatial distribution of correlation coefficients of IOD index of May to November with HadISST April SST anomalies (ºC) for the period 1982-2018. The correlations significant at 95% using Student's two tailed test are marked with crosses. The rectangular boxes represent the regions which are used as input to the ANN models.

The figure was prepared using The NCAR Command Language (version 6.4.0) [Software]. (2017). Boulder, Colorado: UCAR/NCAR/CISL/TDD. http://dx.doi.org/10.5065/D6WD3XH5

Fig S20.a-g) Spatial distribution of correlation coefficients of IOD index of May to November with OIV2 April SST anomalies (°C) for the period 1982-2018. The correlations significant at 95% using Student's two tailed test are marked with crosses. The rectangular boxes represent the regions which are used as input to the ANN models.

The figure was prepared using The NCAR Command Language (version 6.4.0) [Software]. (2017). Boulder, Colorado: UCAR/NCAR/CISL/TDD. http://dx.doi.org/10.5065/D6WD3XH5

Fig S21.a-g) Spatial distribution of correlation coefficients of IOD index of May to November with 850hPa geopotential height anomalies (m) of April for the period 1949-2018. The correlations significant at 95% using Student's two tailed test are marked with crosses. The rectangular boxes represent the regions which are used as input to the ANN models.

The figure was prepared using The NCAR Command Language (version 6.4.0) [Software]. (2017). Boulder, Colorado: UCAR/NCAR/CISL/TDD. http://dx.doi.org/10.5065/D6WD3XH5

Fig S22.a-g) Spatial distribution of correlation coefficients of IOD index of May to November with 850hPa geopotential height anomalies (m) of April for the period 1982-2018. The correlations significant at 95% using Student's two tailed test are marked with crosses. The rectangular boxes represent the regions which are used as input to the ANN models.

The figure was prepared using The NCAR Command Language (version 6.4.0) [Software]. (2017). Boulder, Colorado: UCAR/NCAR/CISL/TDD. http://dx.doi.org/10.5065/D6WD3XH5

Fig S23.a-g) Spatial distribution of correlation coefficients of IOD index of May to November with 200hPa geopotential height anomalies (m) of April for the period 1949-2018. The correlations significant at 95% using Student's two tailed test are marked with crosses. The rectangular boxes represent the regions which are used as input to the ANN models.

The figure was prepared using The NCAR Command Language (version 6.4.0) [Software]. (2017). Boulder, Colorado: UCAR/NCAR/CISL/TDD. http://dx.doi.org/10.5065/D6WD3XH5

Fig S24.a-g) Spatial distribution of correlation coefficients of IOD index of May to November with 200hPa geopotential height anomalies (m) of April for the period 1982-2018. The correlations significant at 95% using Student's two tailed test are marked with crosses. The rectangular boxes represent the regions which are used as input to the ANN models.

The figure was prepared using The NCAR Command Language (version 6.4.0) [Software]. (2017). Boulder, Colorado: UCAR/NCAR/CISL/TDD. http://dx.doi.org/10.5065/D6WD3XH5

Fig S25.a-g) Spatial distribution of regression of IOD index of May to November with surface wind anomalies (ms^{-1}) of April for the period 1949-2018. The vectors are significant at 95% using Student's two tailed test.

The figure was prepared using The NCAR Command Language (version 6.4.0) [Software]. (2017). Boulder, Colorado: UCAR/NCAR/CISL/TDD. http://dx.doi.org/10.5065/D6WD3XH5

Fig S26.a-g) Spatial distribution of regression of IOD index of May to November with surface wind anomalies (ms^{-1}) of April for the period 1982-2018. The vectors are significant at 95% using Student's two tailed test.

The figure was prepared using The NCAR Command Language (version 6.4.0) [Software]. (2017). Boulder, Colorado: UCAR/NCAR/CISL/TDD. http://dx.doi.org/10.5065/D6WD3XH5

Fig S27. Spatial distribution of correlation coefficients of observed IOD index of May-November with the HadISST anomalies for the period 1982-2018. The correlations significant at 95% using Student's two tailed test are marked with crosses.

The figure was prepared using The NCAR Command Language (version 6.4.0) [Software]. (2017). Boulder, Colorado: UCAR/NCAR/CISL/TDD. http://dx.doi.org/10.5065/D6WD3XH5

Fig S28. Spatial distribution of correlation coefficients of observed IOD index of May-November with ensemble mean May-November SST anomalies of February initialized NMME models for the period 1982-2018. The correlations significant at 95% using Student's two tailed test are marked with crosses.

The figure was prepared using The NCAR Command Language (version 6.4.0) [Software]. (2017). Boulder, Colorado: UCAR/NCAR/CISL/TDD. http://dx.doi.org/10.5065/D6WD3XH5

Fig S29. Spatial distribution of correlation coefficients of observed IOD index of May-November with ensemble mean May-November SST anomalies of March initialized NMME

models for the period 1982-2018. The correlations significant at 95% using Student's two tailed test are marked with crosses.

The figure was prepared using The NCAR Command Language (version 6.4.0) [Software]. (2017). Boulder, Colorado: UCAR/NCAR/CISL/TDD. http://dx.doi.org/10.5065/D6WD3XH5

Fig S30. Spatial distribution of correlation coefficients of observed IOD index of May-November with ensemble mean May-November SST anomalies of April initialized NMME models for the period 1982-2018. The correlations significant at 95% using Student's two tailed test are marked with crosses.

The figure was prepared using The NCAR Command Language (version 6.4.0) [Software]. (2017). Boulder, Colorado: UCAR/NCAR/CISL/TDD. http://dx.doi.org/10.5065/D6WD3XH5

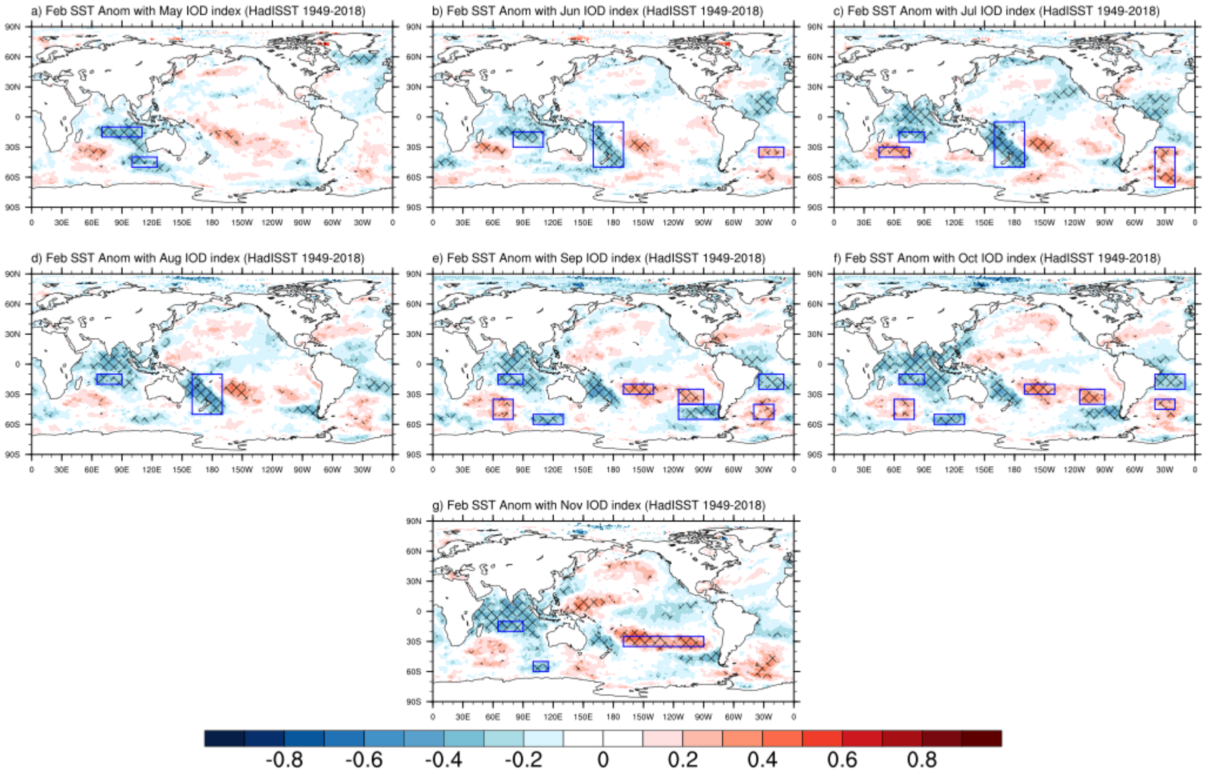

Fig S1.a-g) Spatial distribution of correlation coefficients of IOD index of May to November with HadISST February SST anomalies (ºC) for the period 1949-2018. The correlations significant at 95% using Student's two tailed test are marked with crosses. The rectangular boxes represent the regions which are used as input to the ANN models.

The figure was prepared using The NCAR Command Language (version 6.4.0) [Software]. (2017). Boulder, Colorado: UCAR/NCAR/CISL/TDD. http://dx.doi.org/10.5065/D6WD3XH5

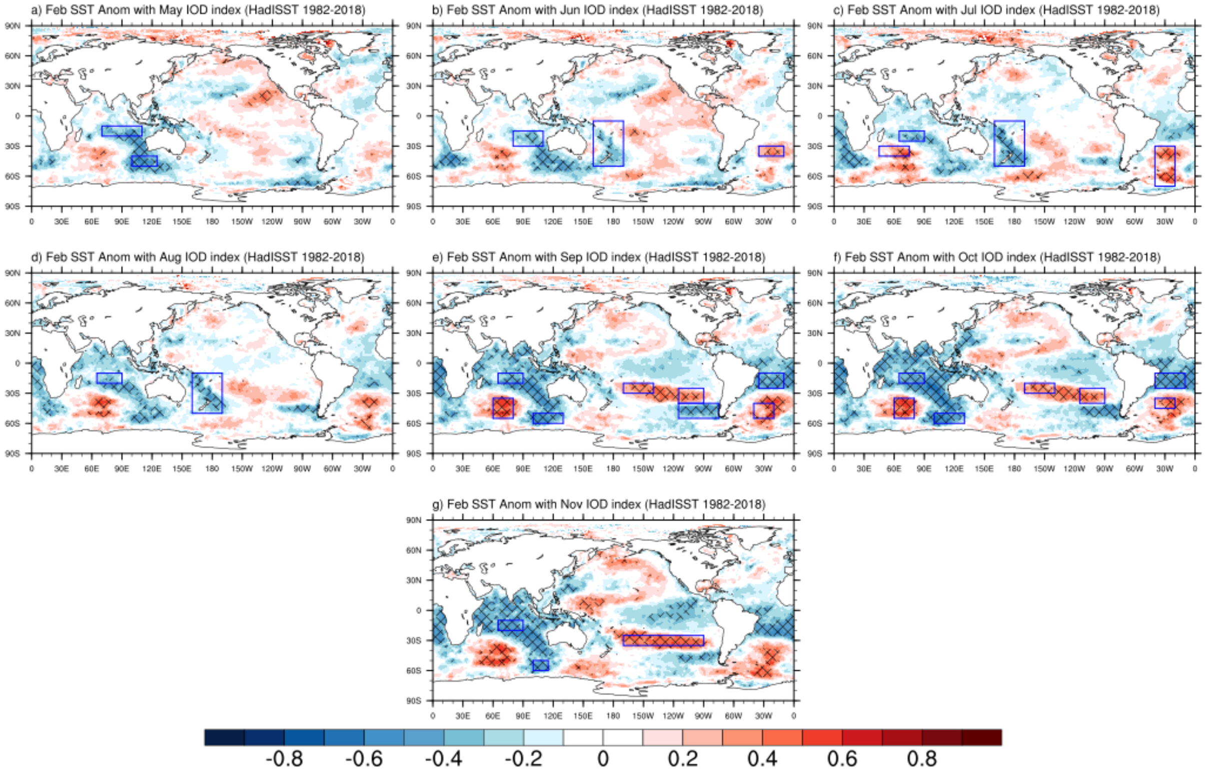

Fig S2.a-g) Spatial distribution of correlation coefficients of IOD index of May to November with HadISST February SST anomalies for the period 1982-2018. The correlations significant at 95% using Student's two tailed test are marked with crosses. The rectangular boxes represent the regions which are used as input to the ANN models.

The figure was prepared using The NCAR Command Language (version 6.4.0) [Software]. (2017). Boulder, Colorado: UCAR/NCAR/CISL/TDD. http://dx.doi.org/10.5065/D6WD3XH5

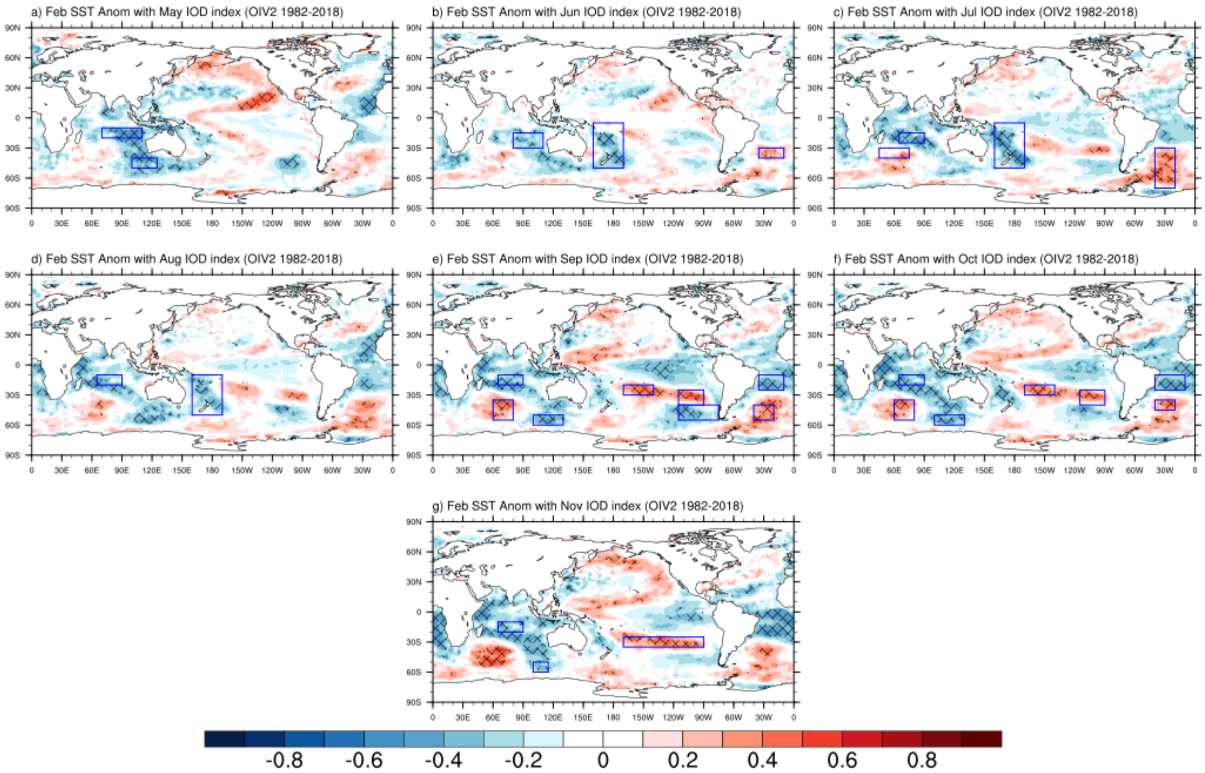

Fig S3.a-g) Spatial distribution of correlation coefficients of IOD index of May to November with OIV2 February SST anomalies for the period 1982-2018. The correlations significant at 95% using Student's two tailed test are marked with crosses. The rectangular boxes represent the regions which are used as input to the ANN models.

The figure was prepared using The NCAR Command Language (version 6.4.0) [Software]. (2017). Boulder, Colorado: UCAR/NCAR/CISL/TDD. http://dx.doi.org/10.5065/D6WD3XH5

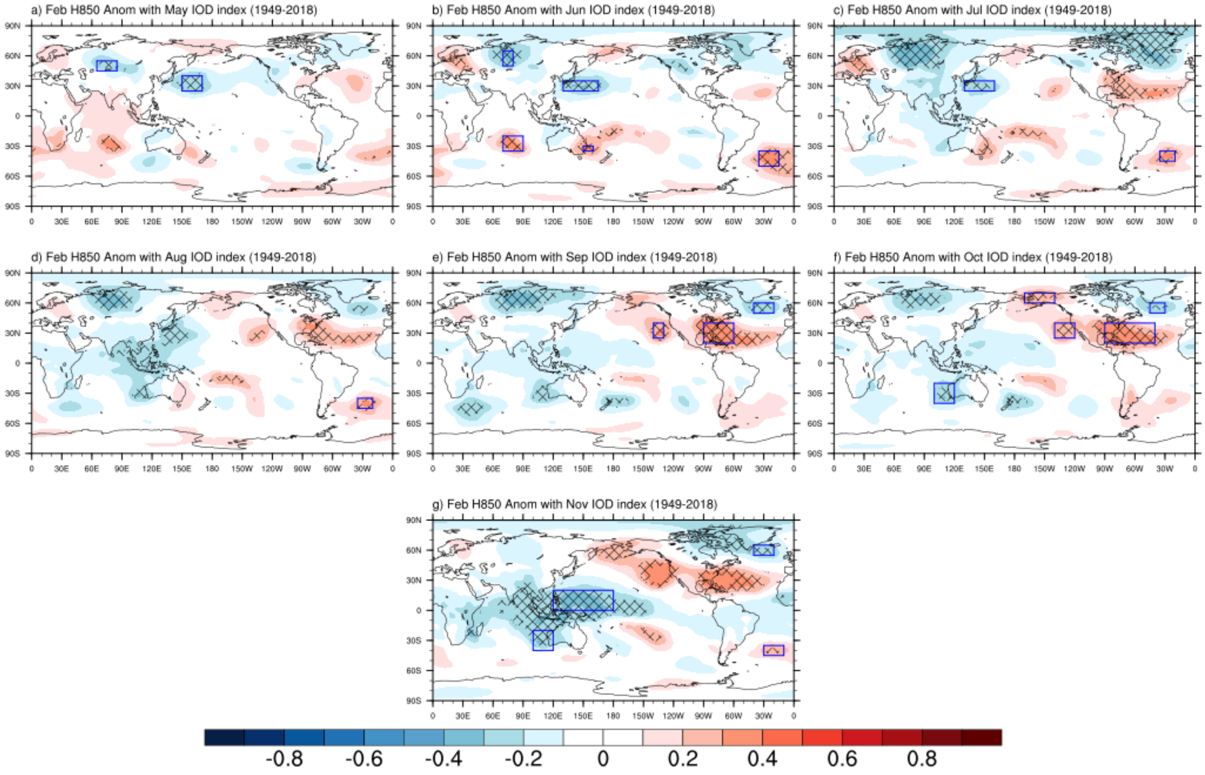

Fig S4.a-g) Spatial distribution of correlation coefficients of IOD index of May to November with 850hPa geopotential height anomalies (m) of February SST anomalies for the period 1949-2018. The correlations significant at 95% using Student's two tailed test are marked with crosses. The rectangular boxes represent the regions which are used as input to the ANN models.

The figure was prepared using The NCAR Command Language (version 6.4.0) [Software]. (2017). Boulder, Colorado: UCAR/NCAR/CISL/TDD. http://dx.doi.org/10.5065/D6WD3XH5

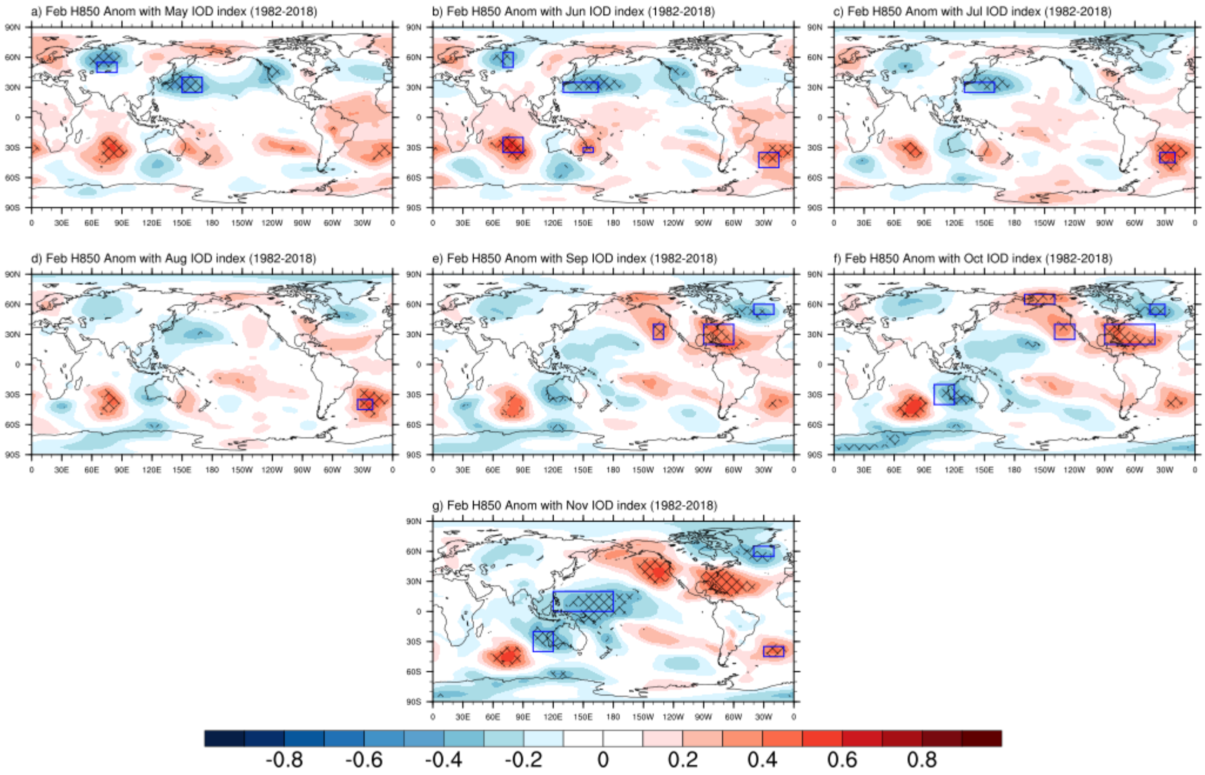

Fig S5.a-g) Spatial distribution of correlation coefficients of IOD index of May to November with 850hPa geopotential height anomalies (m) of February SST anomalies for the period 1982-2018. The correlations significant at 95% using Student's two tailed test are marked with crosses. The rectangular boxes represent the regions which are used as input to the ANN models.

The figure was prepared using The NCAR Command Language (version 6.4.0) [Software]. (2017). Boulder, Colorado: UCAR/NCAR/CISL/TDD. http://dx.doi.org/10.5065/D6WD3XH5

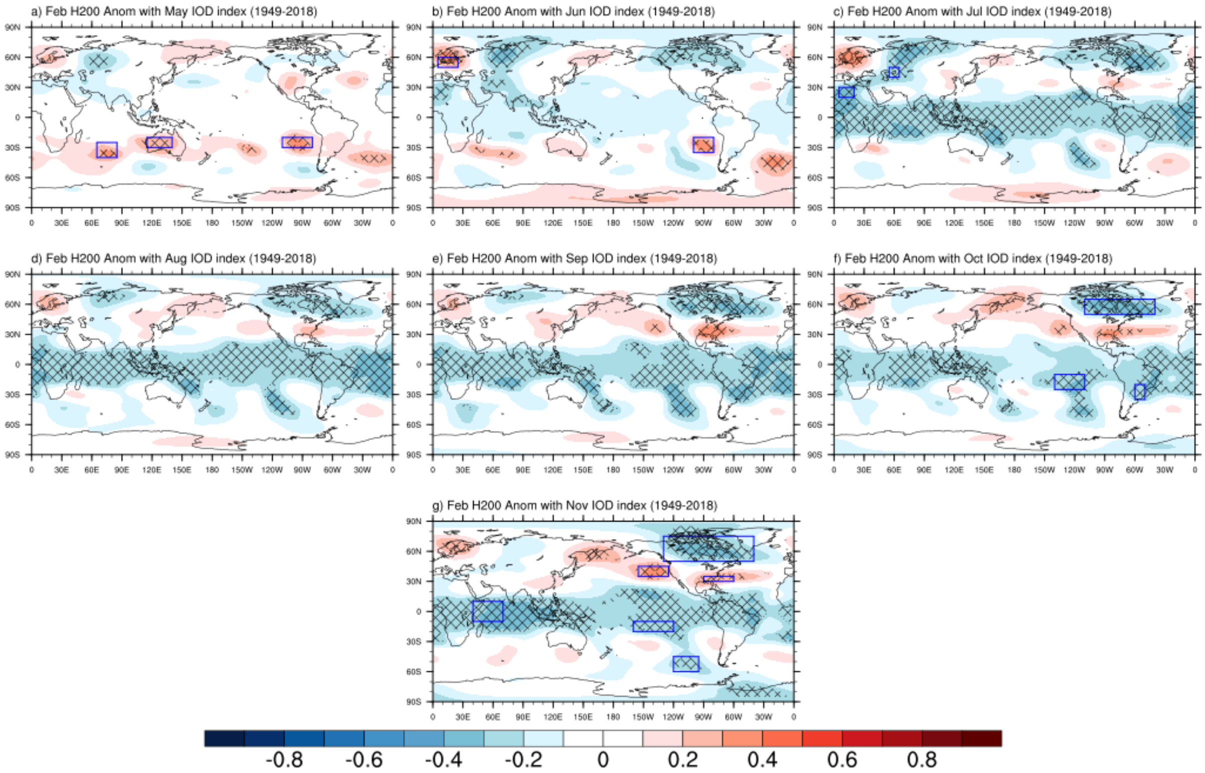

Fig S6.a-g) Spatial distribution of correlation coefficients of IOD index of May to November with 200hPa geopotential height anomalies (m) of February SST anomalies for the period 1949-2018. The correlations significant at 95% using Student's two tailed test are marked with crosses. The rectangular boxes represent the regions which are used as input to the ANN models.

The figure was prepared using The NCAR Command Language (version 6.4.0) [Software]. (2017). Boulder, Colorado: UCAR/NCAR/CISL/TDD. http://dx.doi.org/10.5065/D6WD3XH5

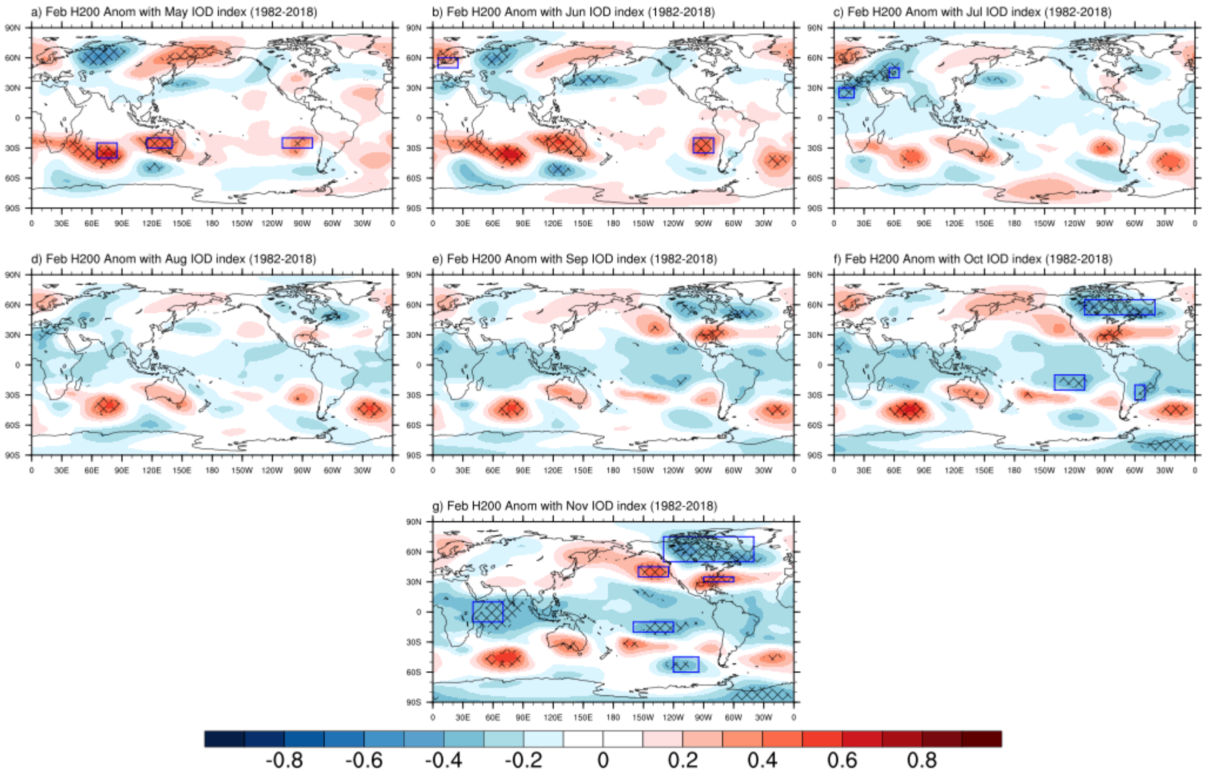

Fig S7.a-g) Spatial distribution of correlation coefficients of IOD index of May to November with 200hPa geopotential height anomalies (m) of February SST anomalies for the period 1982-2018. The correlations significant at 95% using Student's two tailed test are marked with crosses. The rectangular boxes represent the regions which are used as input to the ANN models.

The figure was prepared using The NCAR Command Language (version 6.4.0) [Software]. (2017). Boulder, Colorado: UCAR/NCAR/CISL/TDD. http://dx.doi.org/10.5065/D6WD3XH5

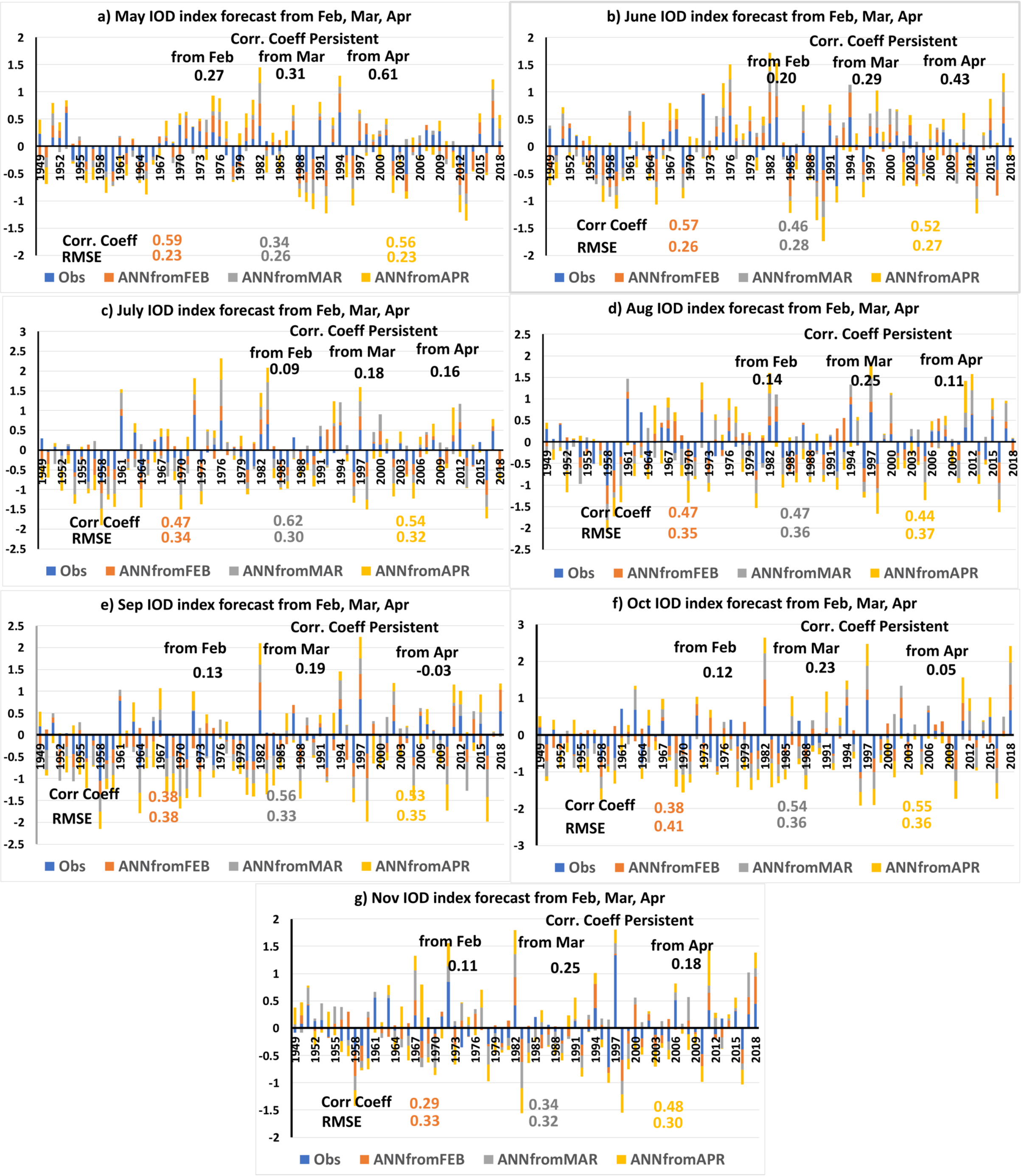

Fig S8a-g. Ensemble mean of 500 members forecast of IOD index for the months of May to November from February, March and April initial conditions using ANN models for the period 1949 to 2018.

The panels were prepared using Microsoft EXCEL 2016 and merged with ImageMagick software (version 6.7.2-7) (https://imagemagick.org/)

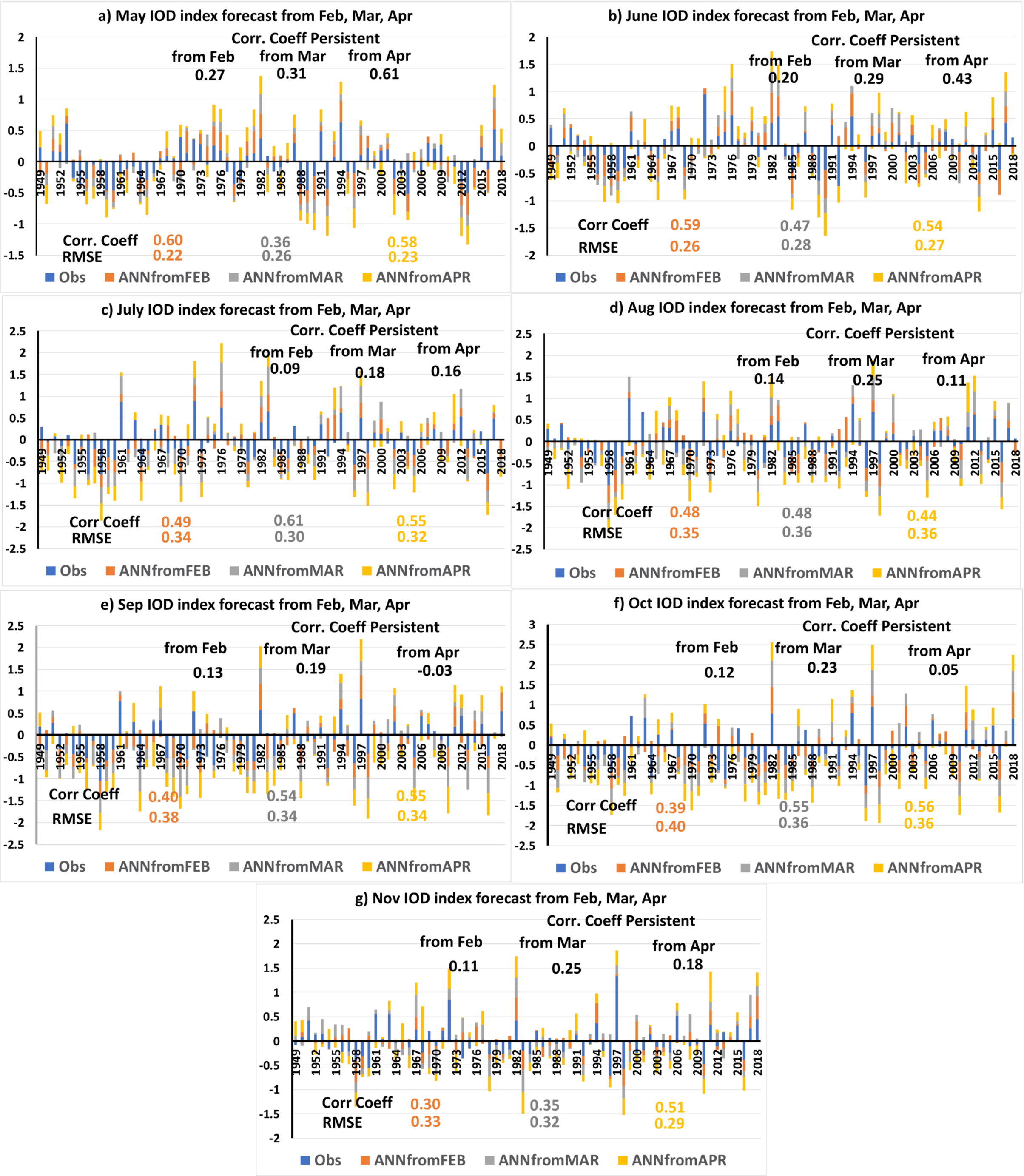

Fig S9a-g. Ensemble mean of 500 members forecast of IOD index for the months of May to November from February, March and April initial conditions using ANN models for the period 1949 to 2018 using 70 % of each sample for training.

The panels were prepared using Microsoft EXCEL 2016 and merged with ImageMagick software (version 6.7.2-7) (https://imagemagick.org/)

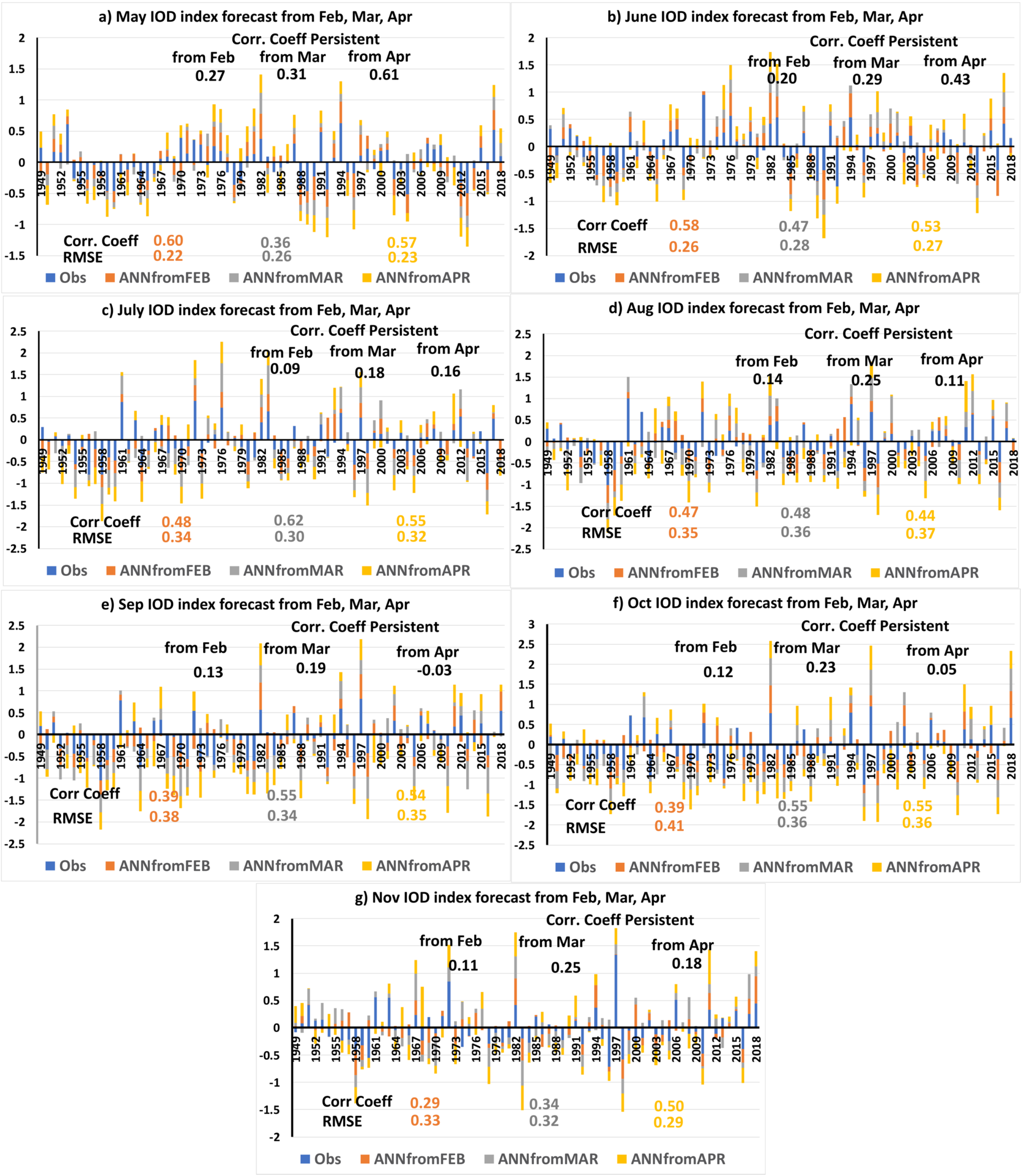

Fig S10a-g. Ensemble mean of 500 members forecast of IOD index for the months of May to November from February, March and April initial conditions using ANN models for the period 1949 to 2018 using 80 % of each sample for training.

The panels were prepared using Microsoft EXCEL 2016 and merged with ImageMagick software (version 6.7.2-7) (https://imagemagick.org/)

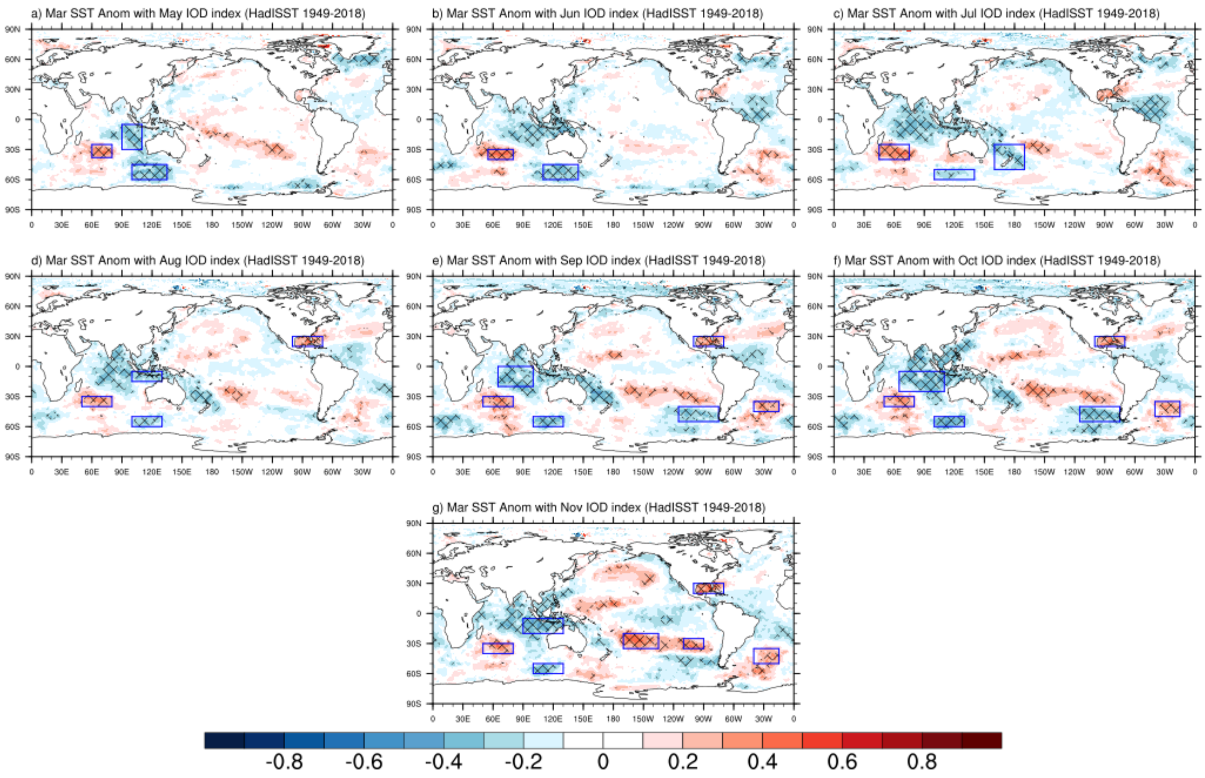

Fig S11.a-g) Spatial distribution of correlation coefficients of IOD index of May to November with HadISST March SST anomalies (°C) for the period 1949-2018. The correlations significant at 95% using Student's two tailed test are marked with crosses. The rectangular boxes represent the regions which are used as input to the ANN models.

The figure was prepared using The NCAR Command Language (version 6.4.0) [Software]. (2017). Boulder, Colorado: UCAR/NCAR/CISL/TDD. http://dx.doi.org/10.5065/D6WD3XH5

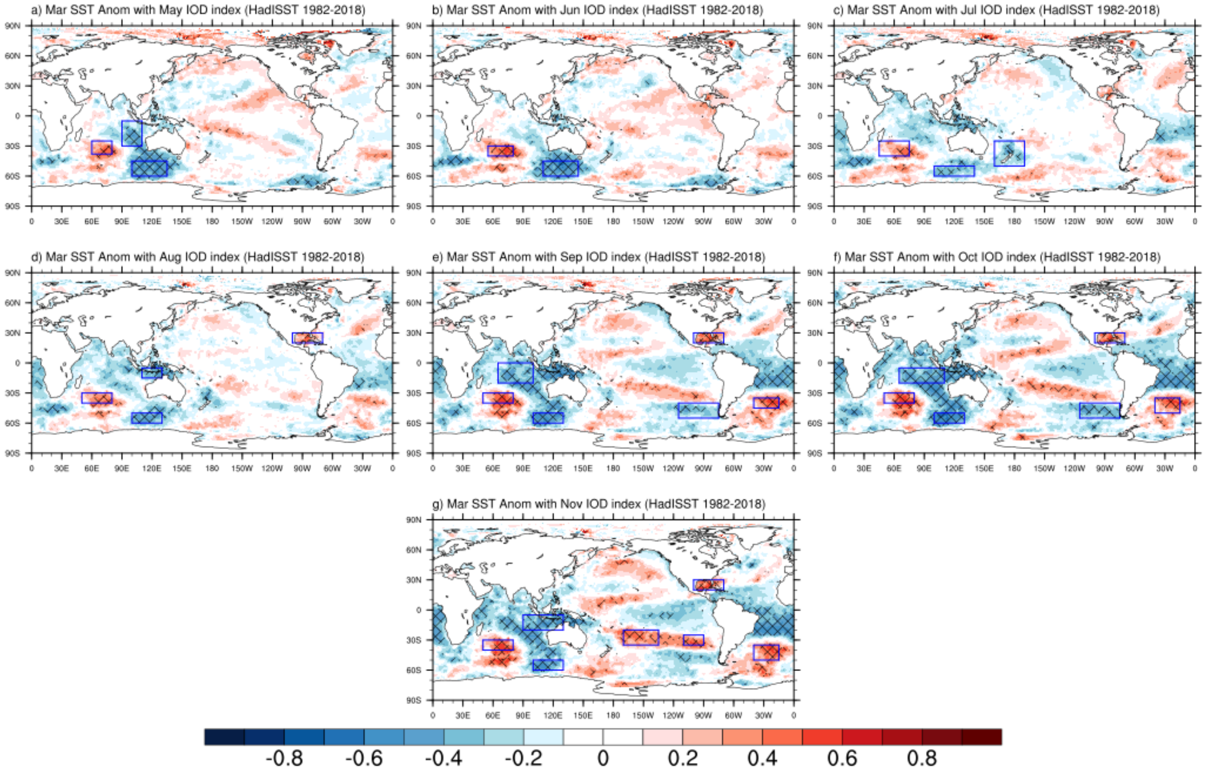

Fig S12.a-g) Spatial distribution of correlation coefficients of IOD index of May to November with HadISST March SST anomalies (ºC) for the period 1982-2018. The correlations significant at 95% using Student's two tailed test are marked with crosses. The rectangular boxes represent the regions which are used as input to the ANN models.

The figure was prepared using The NCAR Command Language (version 6.4.0) [Software]. (2017). Boulder, Colorado: UCAR/NCAR/CISL/TDD. http://dx.doi.org/10.5065/D6WD3XH5

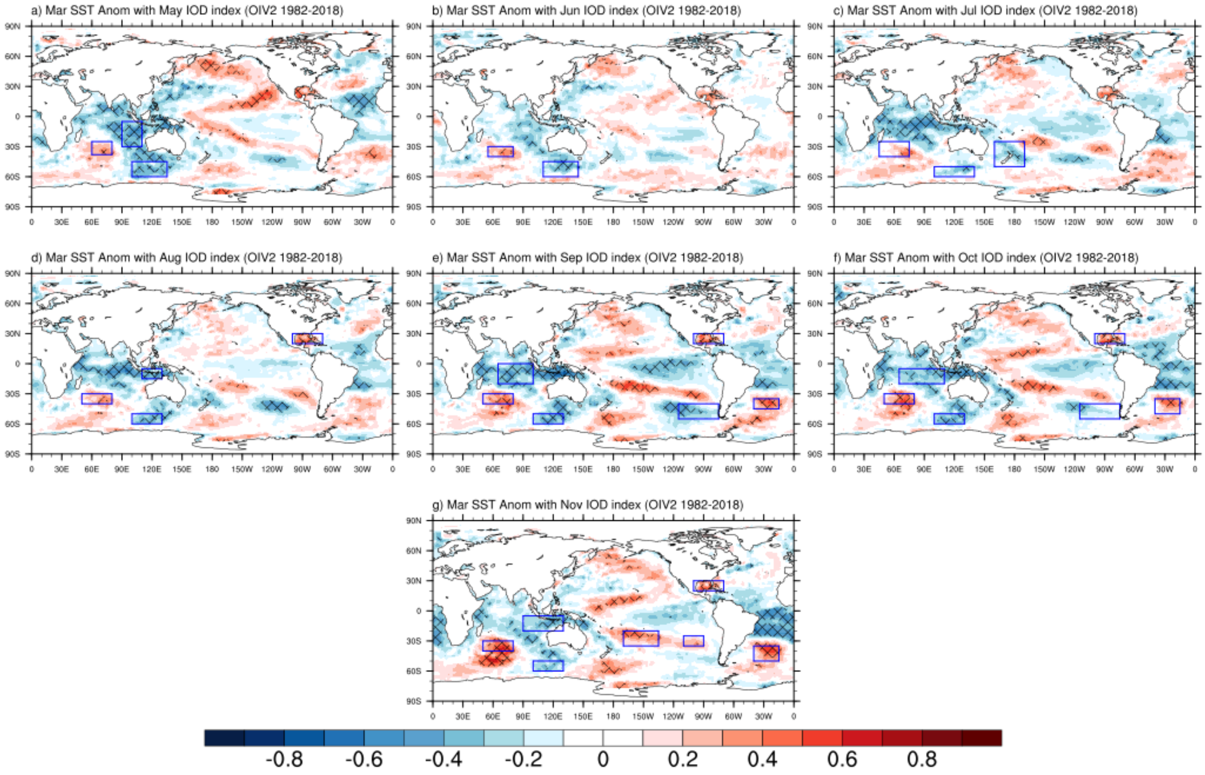

Fig S13.a-g) Spatial distribution of correlation coefficients of IOD index of May to November with OIV2 March SST anomalies (°C) for the period 1982-2018. The correlations significant at 95% using Student's two tailed test are marked with crosses. The rectangular boxes represent the regions which are used as input to the ANN models.

The figure was prepared using The NCAR Command Language (version 6.4.0) [Software]. (2017). Boulder, Colorado: UCAR/NCAR/CISL/TDD. http://dx.doi.org/10.5065/D6WD3XH5

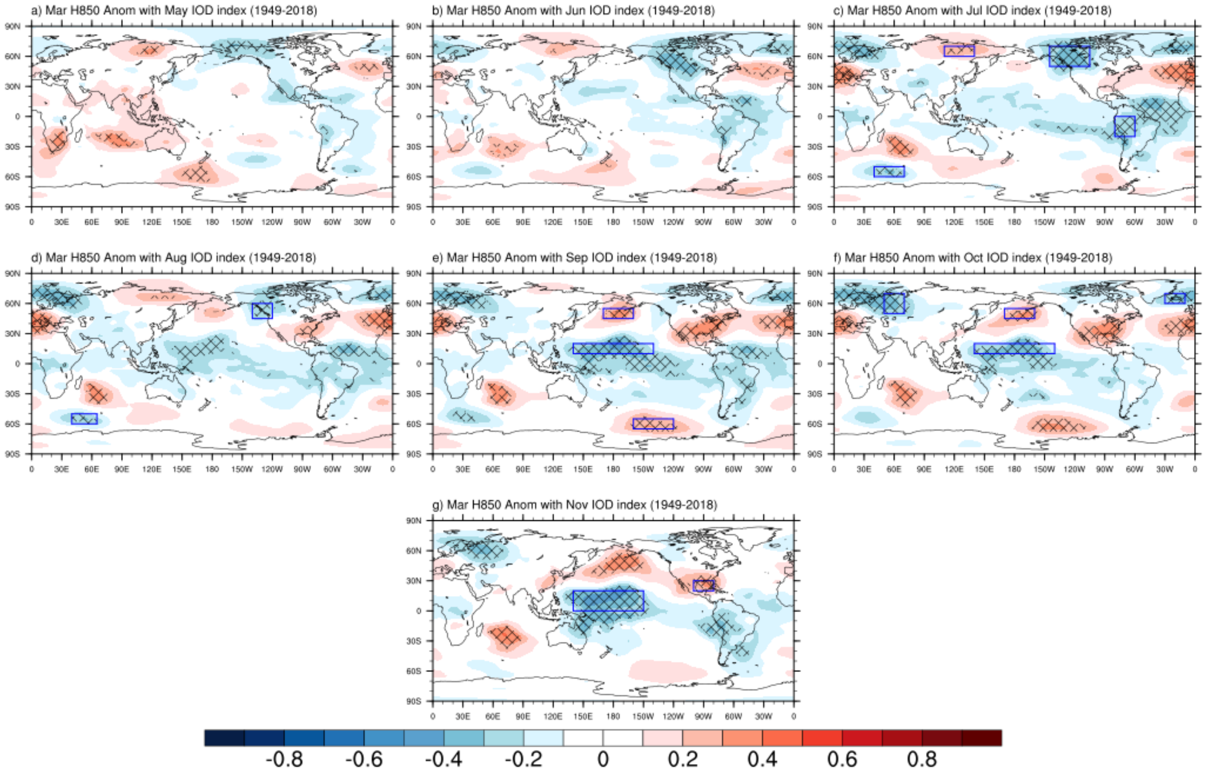

Fig S14.a-g) Spatial distribution of correlation coefficients of IOD index of May to November with 850hPa geopotential height anomalies (m) of March for the period 1949-2018. The correlations significant at 95% using Student's two tailed test are marked with crosses. The rectangular boxes represent the regions which are used as input to the ANN models.

The figure was prepared using The NCAR Command Language (version 6.4.0) [Software]. (2017). Boulder, Colorado: UCAR/NCAR/CISL/TDD. http://dx.doi.org/10.5065/D6WD3XH5

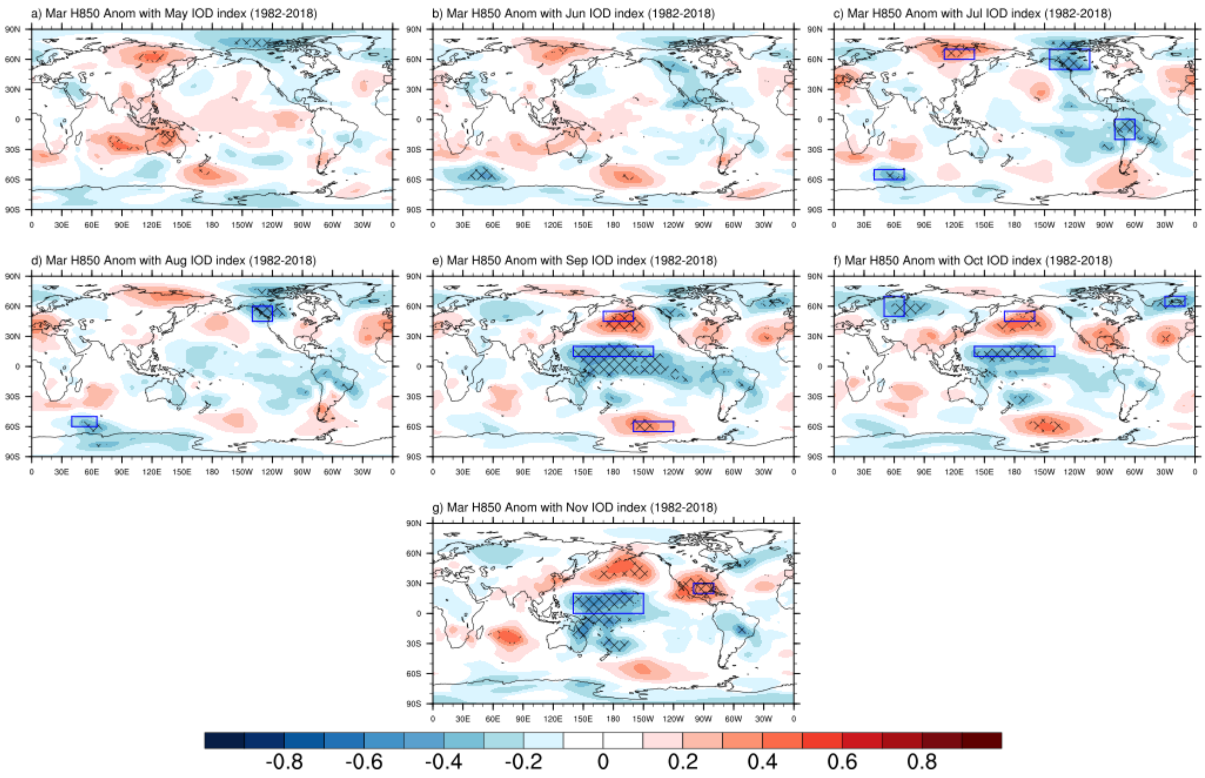

Fig S15.a-g) Spatial distribution of correlation coefficients of IOD index of May to November with 850hPa geopotential height anomalies (m) of March for the period 1982-2018. The correlations significant at 95% using Student's two tailed test are marked with crosses. The rectangular boxes represent the regions which are used as input to the ANN models.

The figure was prepared using The NCAR Command Language (version 6.4.0) [Software]. (2017). Boulder, Colorado: UCAR/NCAR/CISL/TDD. http://dx.doi.org/10.5065/D6WD3XH5

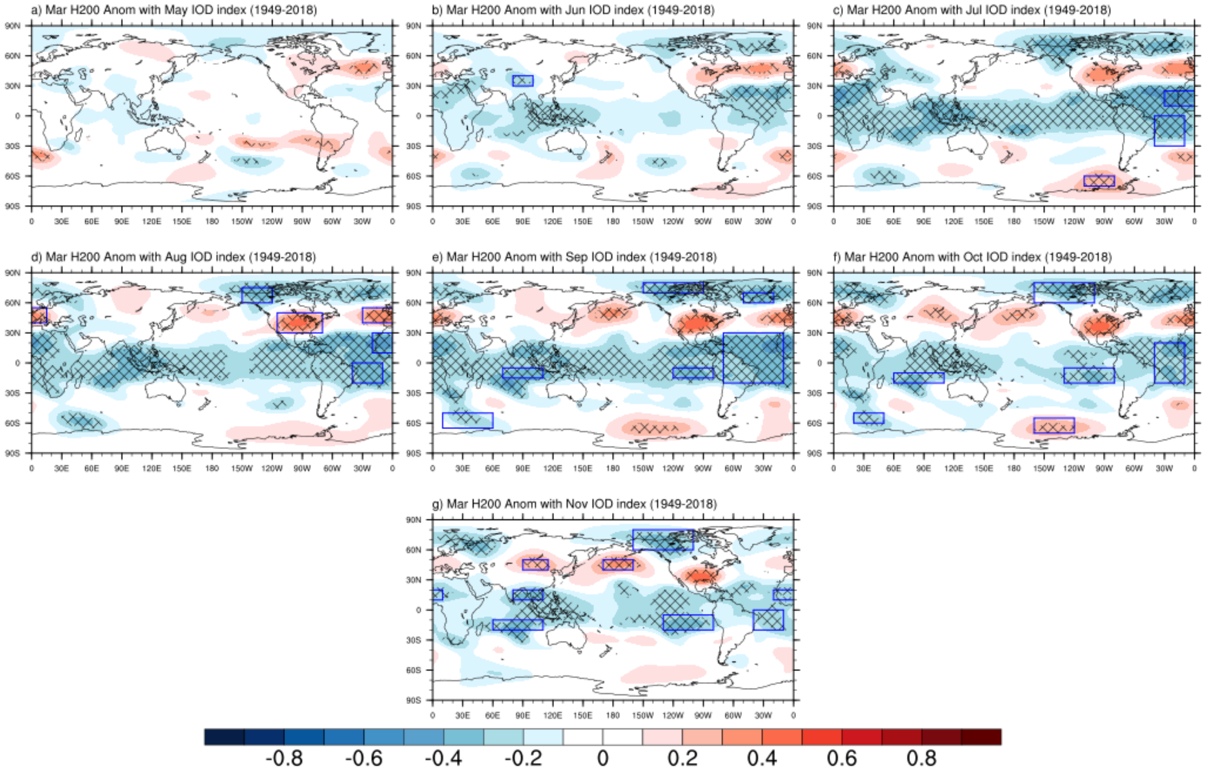

Fig S16.a-g) Spatial distribution of correlation coefficients of IOD index of May to November with 200hPa geopotential height anomalies (m) of March for the period 1949-2018. The correlations significant at 95% using Student's two tailed test are marked with crosses. The rectangular boxes represent the regions which are used as input to the ANN models.

The figure was prepared using The NCAR Command Language (version 6.4.0) [Software]. (2017). Boulder, Colorado: UCAR/NCAR/CISL/TDD. http://dx.doi.org/10.5065/D6WD3XH5

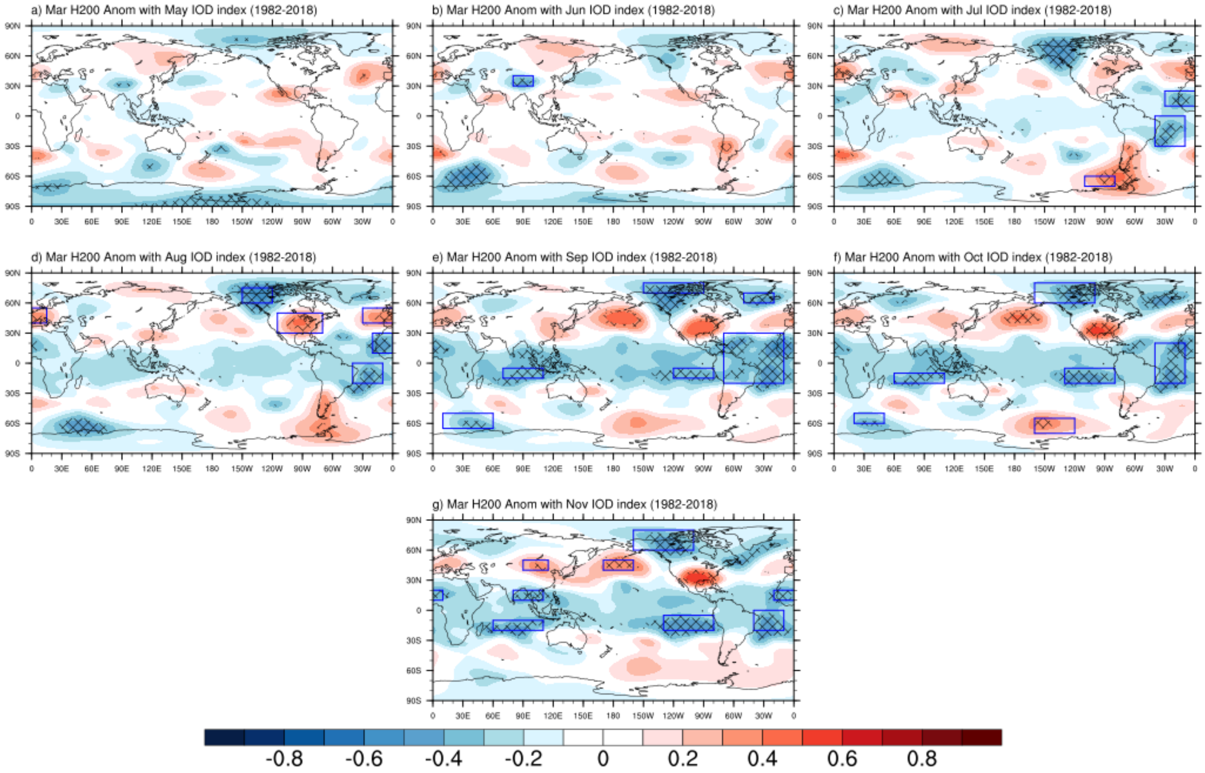

Fig S17.a-g) Spatial distribution of correlation coefficients of IOD index of May to November with 200hPa geopotential height anomalies (m) of March for the period 1982-2018. The correlations significant at 95% using Student's two tailed test are marked with crosses. The rectangular boxes represent the regions which are used as input to the ANN models.

The figure was prepared using The NCAR Command Language (version 6.4.0) [Software]. (2017). Boulder, Colorado: UCAR/NCAR/CISL/TDD. http://dx.doi.org/10.5065/D6WD3XH5

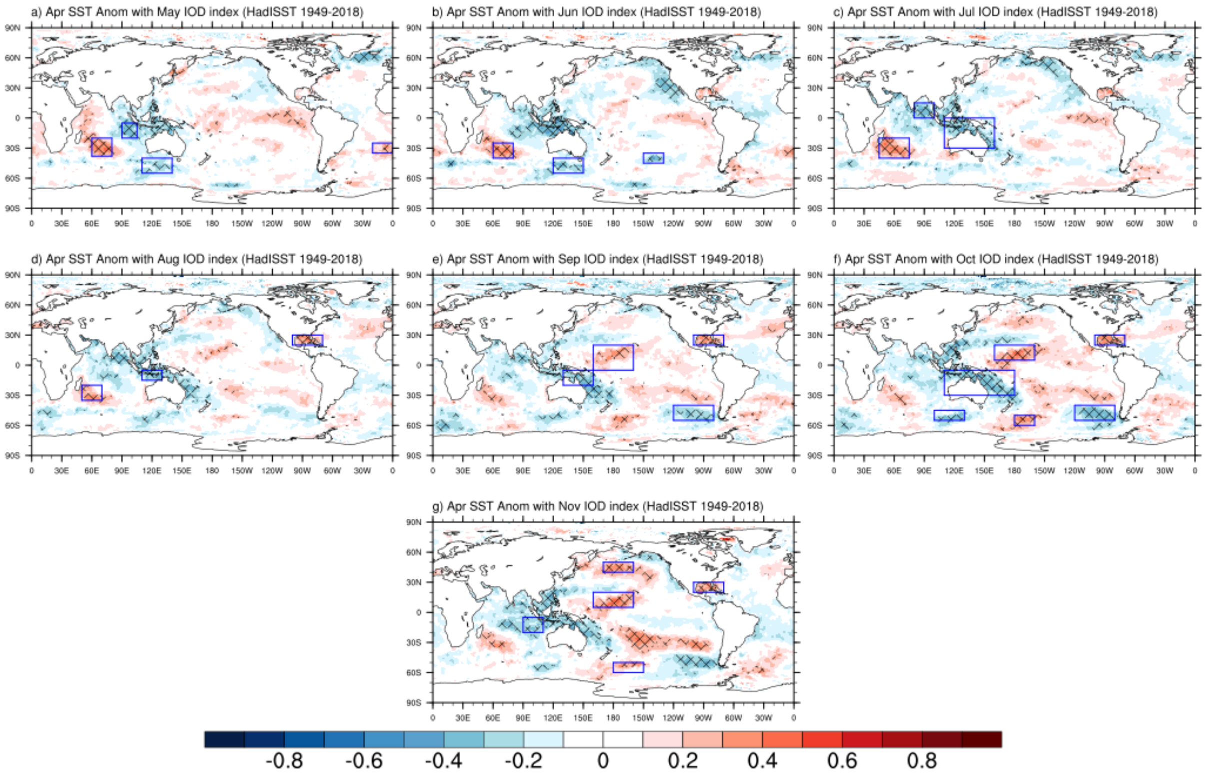

Fig S18.a-g) Spatial distribution of correlation coefficients of IOD index of May to November with HadISST April SST anomalies for the period 1949-2018. The correlations significant at 95% using Student's two tailed test are marked with crosses. The rectangular boxes represent the regions which are used as input to the ANN models.

The figure was prepared using The NCAR Command Language (version 6.4.0) [Software]. (2017). Boulder, Colorado: UCAR/NCAR/CISL/TDD. http://dx.doi.org/10.5065/D6WD3XH5

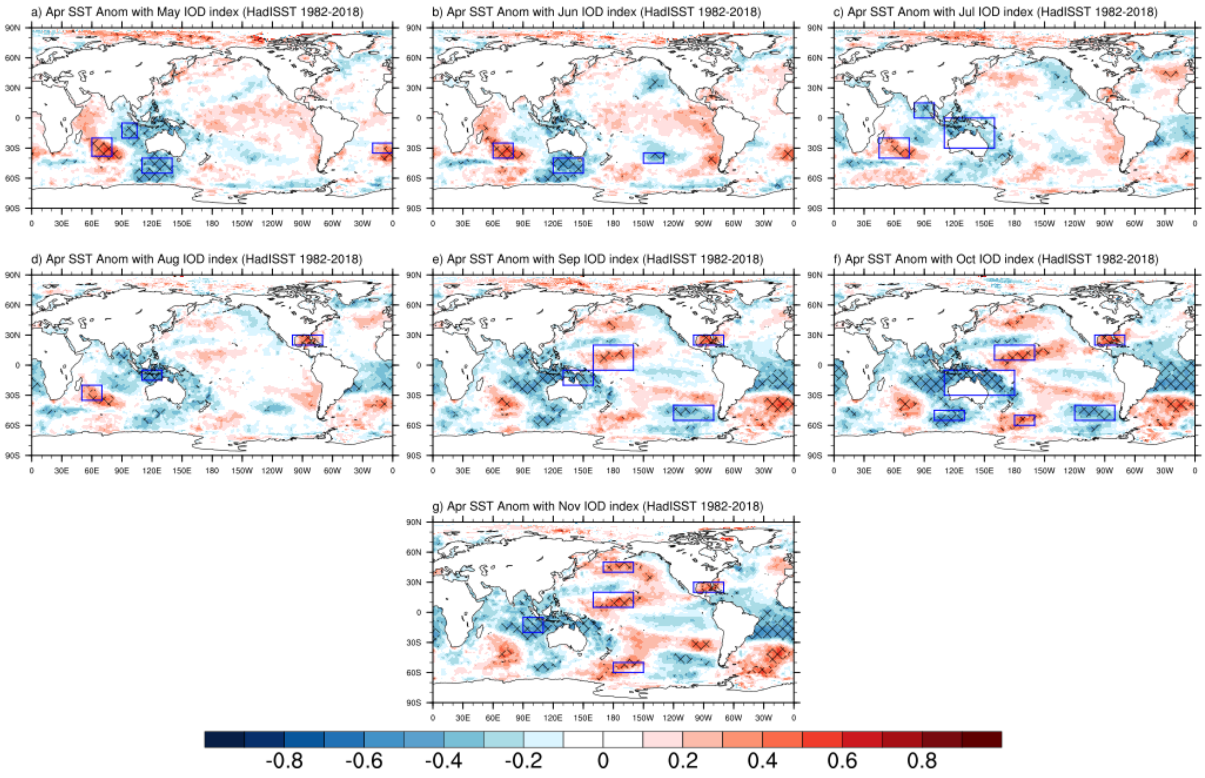

Fig S19.a-g) Spatial distribution of correlation coefficients of IOD index of May to November with HadISST April SST anomalies for the period 1982-2018. The correlations significant at 95% using Student's two tailed test are marked with crosses. The rectangular boxes represent the regions which are used as input to the ANN models.

The figure was prepared using The NCAR Command Language (version 6.4.0) [Software]. (2017). Boulder, Colorado: UCAR/NCAR/CISL/TDD. http://dx.doi.org/10.5065/D6WD3XH5

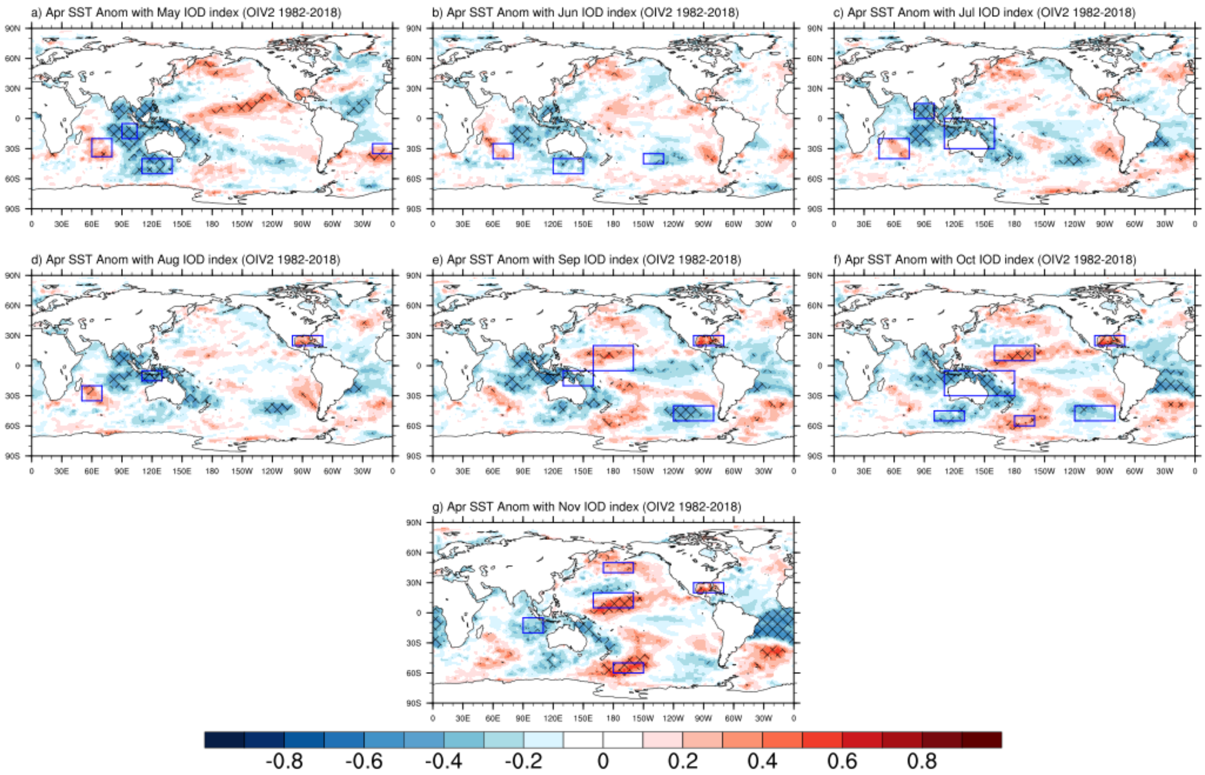

Fig S20.a-g) Spatial distribution of correlation coefficients of IOD index of May to November with OIV2 April SST anomalies for the period 1982-2018. The correlations significant at 95% using Student's two tailed test are marked with crosses. The rectangular boxes represent the regions which are used as input to the ANN models.

The figure was prepared using The NCAR Command Language (version 6.4.0) [Software]. (2017). Boulder, Colorado: UCAR/NCAR/CISL/TDD. http://dx.doi.org/10.5065/D6WD3XH5

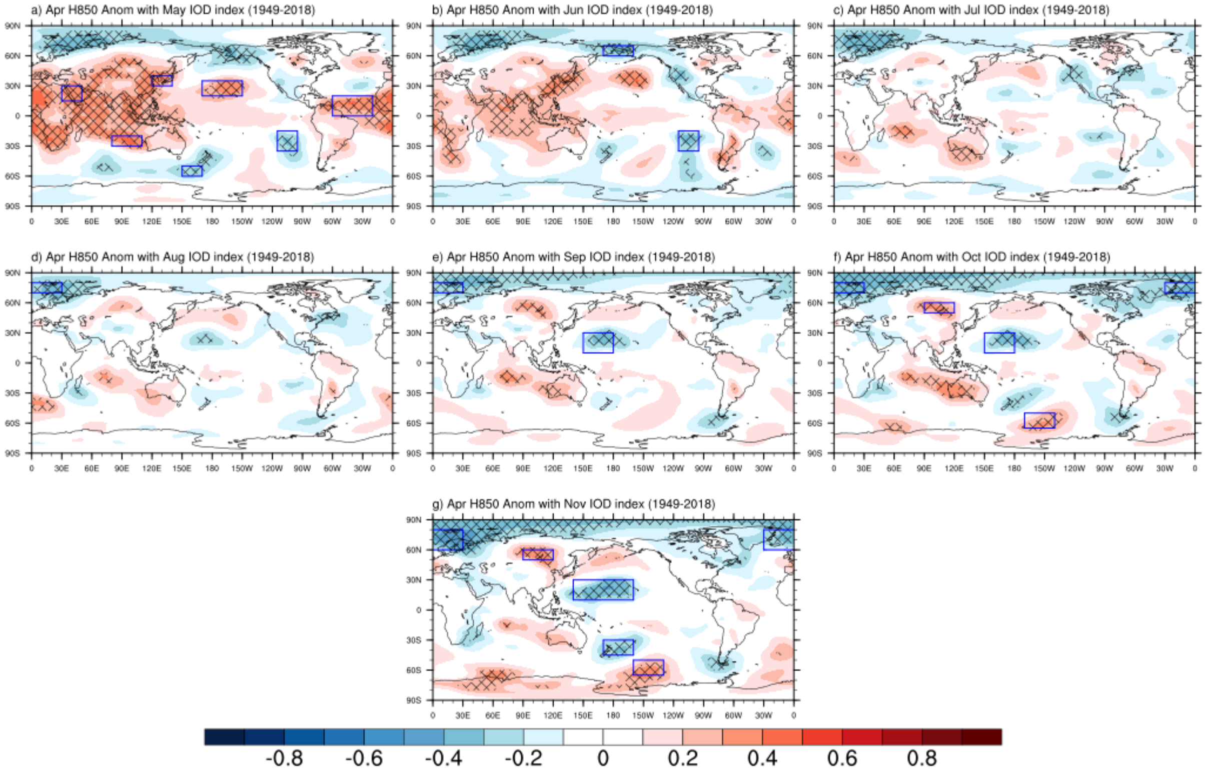

Fig S21.a-g) Spatial distribution of correlation coefficients of IOD index of May to November with 850hPa geopotential height anomalies (m) of April for the period 1949-2018. The correlations significant at 95% using Student's two tailed test are marked with crosses. The rectangular boxes represent the regions which are used as input to the ANN models.

The figure was prepared using The NCAR Command Language (version 6.4.0) [Software]. (2017). Boulder, Colorado: UCAR/NCAR/CISL/TDD. http://dx.doi.org/10.5065/D6WD3XH5

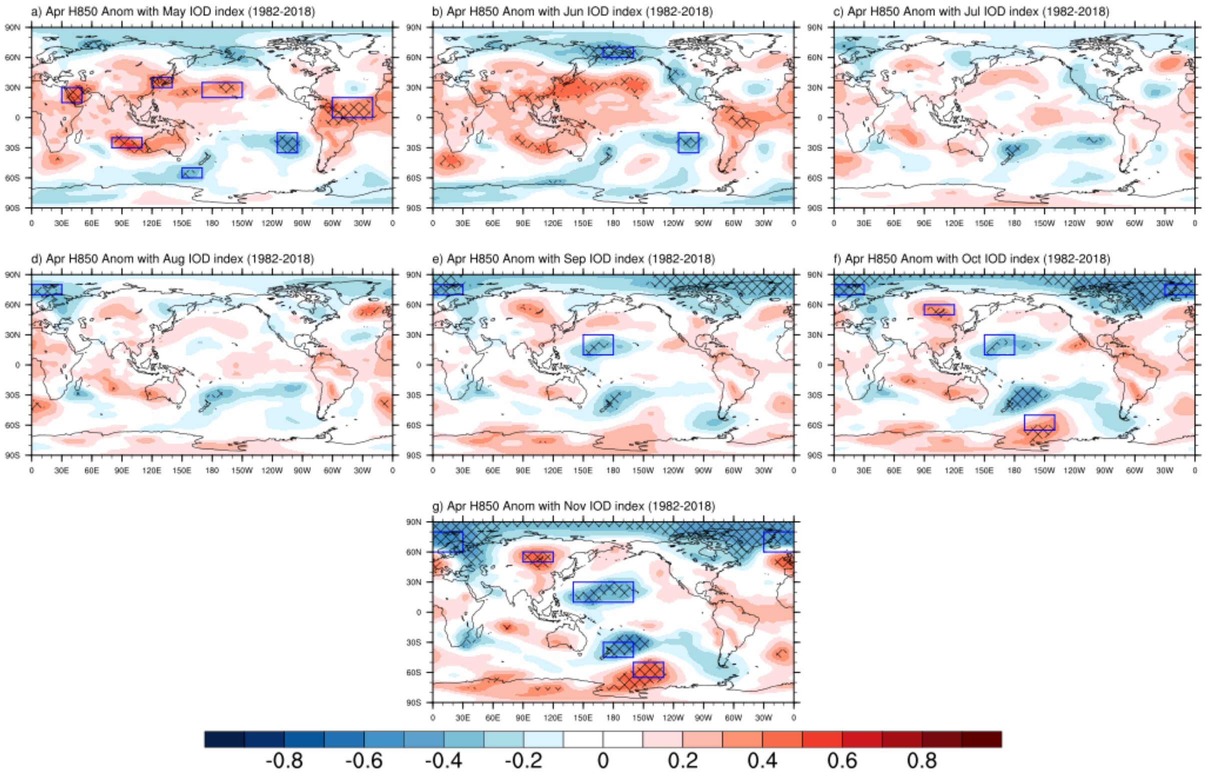

Fig S22.a-g) Spatial distribution of correlation coefficients of IOD index of May to November with 850hPa geopotential height anomalies (m) of April for the period 1982-2018. The correlations significant at 95% using Student's two tailed test are marked with crosses. The rectangular boxes represent the regions which are used as input to the ANN models.

The figure was prepared using The NCAR Command Language (version 6.4.0) [Software]. (2017). Boulder, Colorado: UCAR/NCAR/CISL/TDD. http://dx.doi.org/10.5065/D6WD3XH5

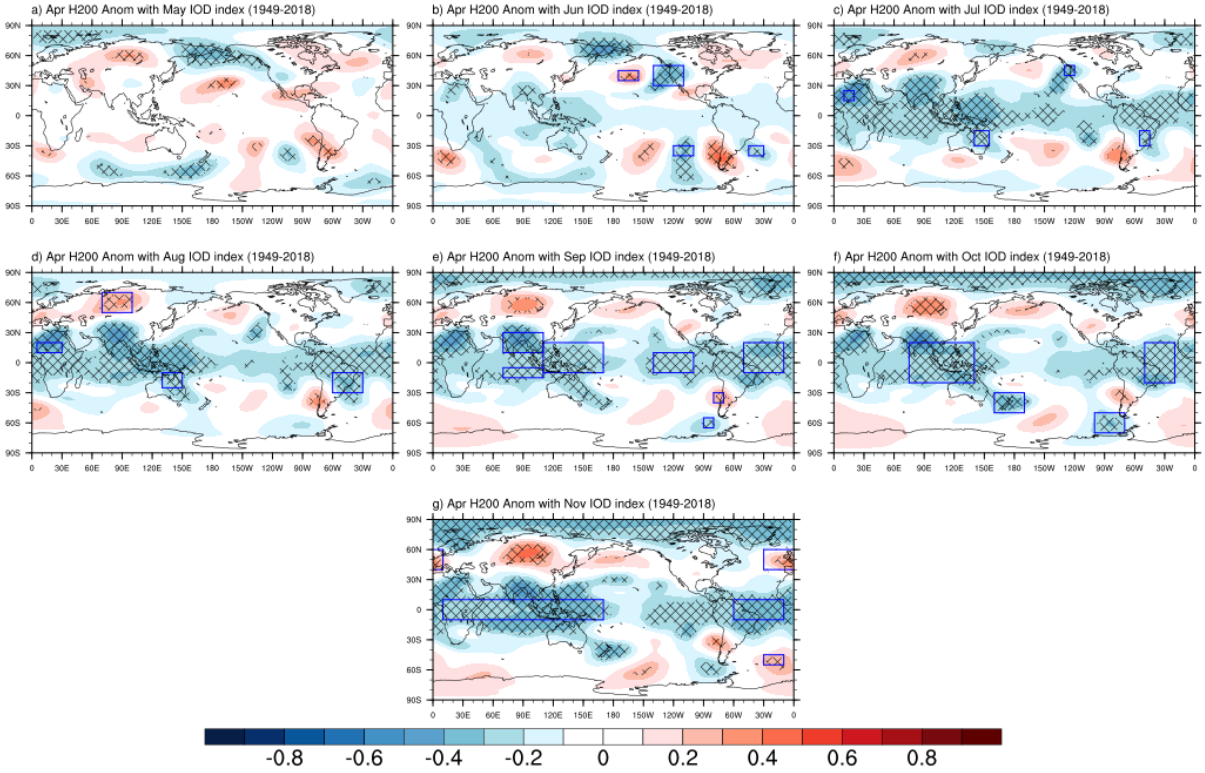

Fig S23.a-g) Spatial distribution of correlation coefficients of IOD index of May to November with 200hPa geopotential height anomalies (m) of April for the period 1949-2018. The correlations significant at 95% using Student's two tailed test are marked with crosses. The rectangular boxes represent the regions which are used as input to the ANN models.

The figure was prepared using The NCAR Command Language (version 6.4.0) [Software]. (2017). Boulder, Colorado: UCAR/NCAR/CISL/TDD. http://dx.doi.org/10.5065/D6WD3XH5

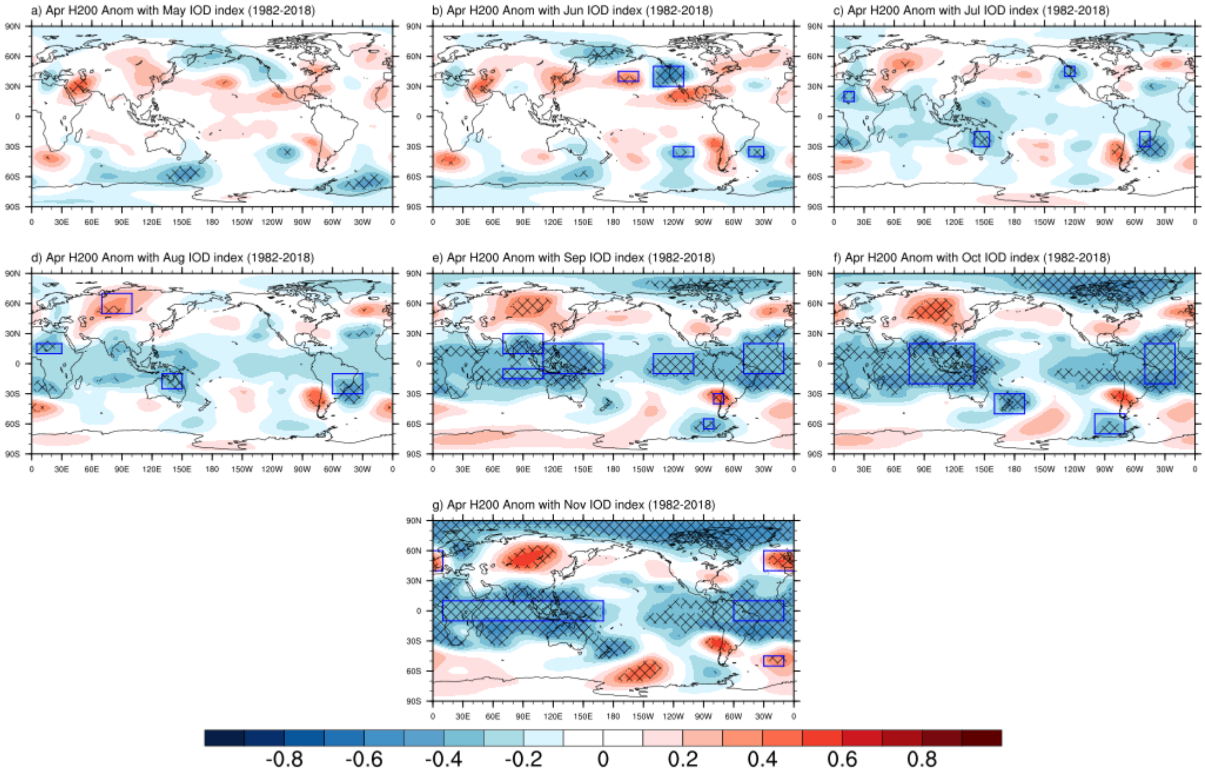

Fig S24.a-g) Spatial distribution of correlation coefficients of IOD index of May to November with 200hPa geopotential height anomalies (m) of April for the period 1982-2018. The correlations significant at 95% using Student's two tailed test are marked with crosses. The rectangular boxes represent the regions which are used as input to the ANN models.

The figure was prepared using The NCAR Command Language (version 6.4.0) [Software]. (2017). Boulder, Colorado: UCAR/NCAR/CISL/TDD. http://dx.doi.org/10.5065/D6WD3XH5

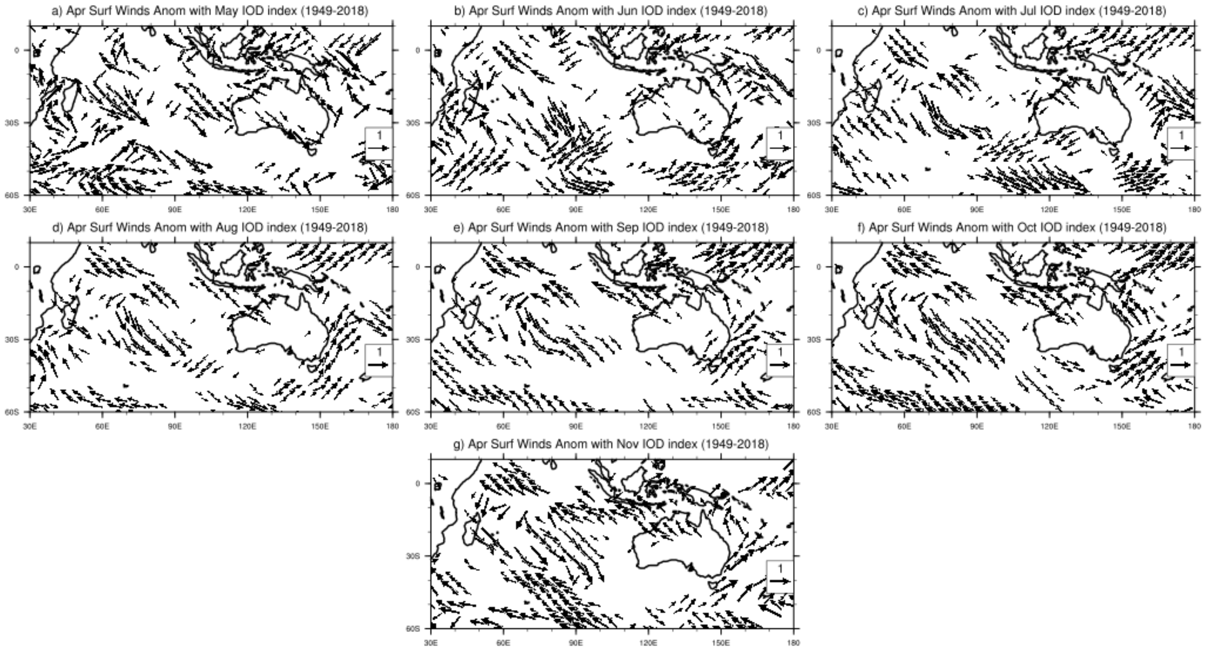

Fig S25.a-g) Spatial distribution of regression of IOD index of May to November with surface wind anomalies (ms^{-1}) of April for the period 1949-2018. The vectors are significant at 95% using Student's two tailed test.

The figure was prepared using The NCAR Command Language (version 6.4.0) [Software]. (2017). Boulder, Colorado: UCAR/NCAR/CISL/TDD. http://dx.doi.org/10.5065/D6WD3XH5

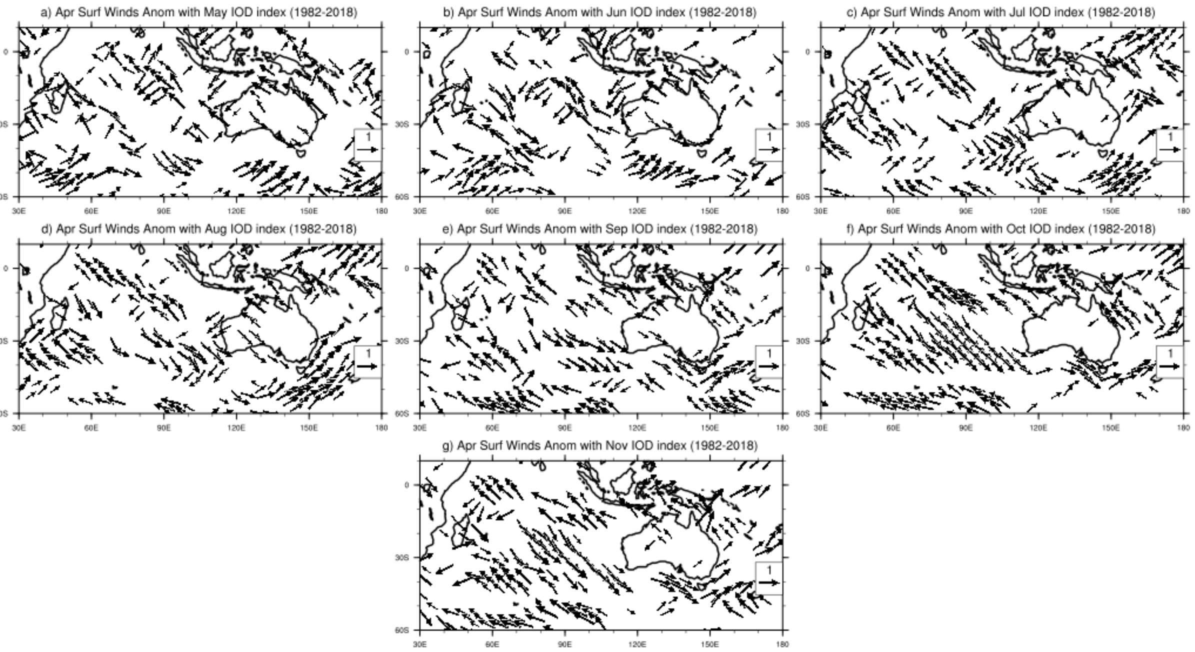

Fig S26.a-g) Spatial distribution of regression of IOD index of May to November with surface wind anomalies (ms^{-1}) of April for the period 1982-2018. The vectors are significant at 95% using Student's two tailed test.

The figure was prepared using The NCAR Command Language (version 6.4.0) [Software]. (2017). Boulder, Colorado: UCAR/NCAR/CISL/TDD. http://dx.doi.org/10.5065/D6WD3XH5

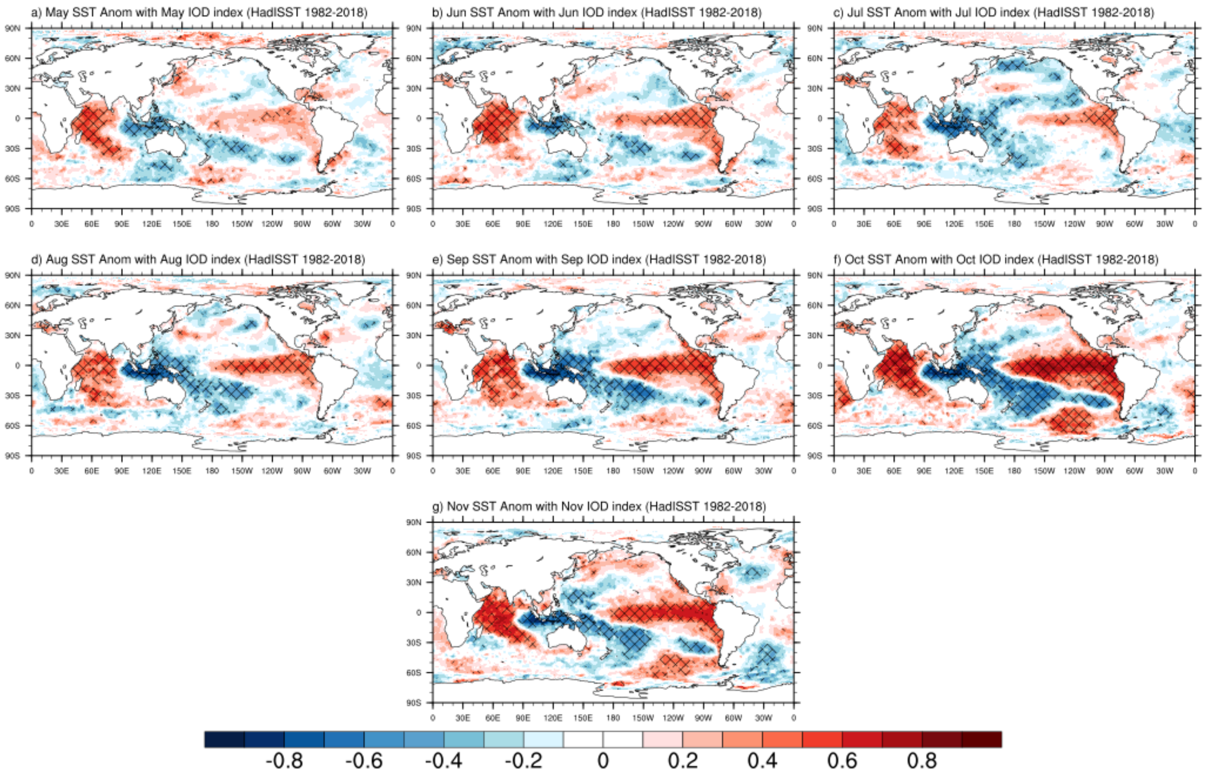

Fig S27. Spatial distribution of correlation coefficients of observed IOD index of May-November with the HadISST anomalies for the period 1982-2018. The correlations significant at 95% using Student's two tailed test are marked with crosses.

The figure was prepared using The NCAR Command Language (version 6.4.0) [Software]. (2017). Boulder, Colorado: UCAR/NCAR/CISL/TDD. http://dx.doi.org/10.5065/D6WD3XH5

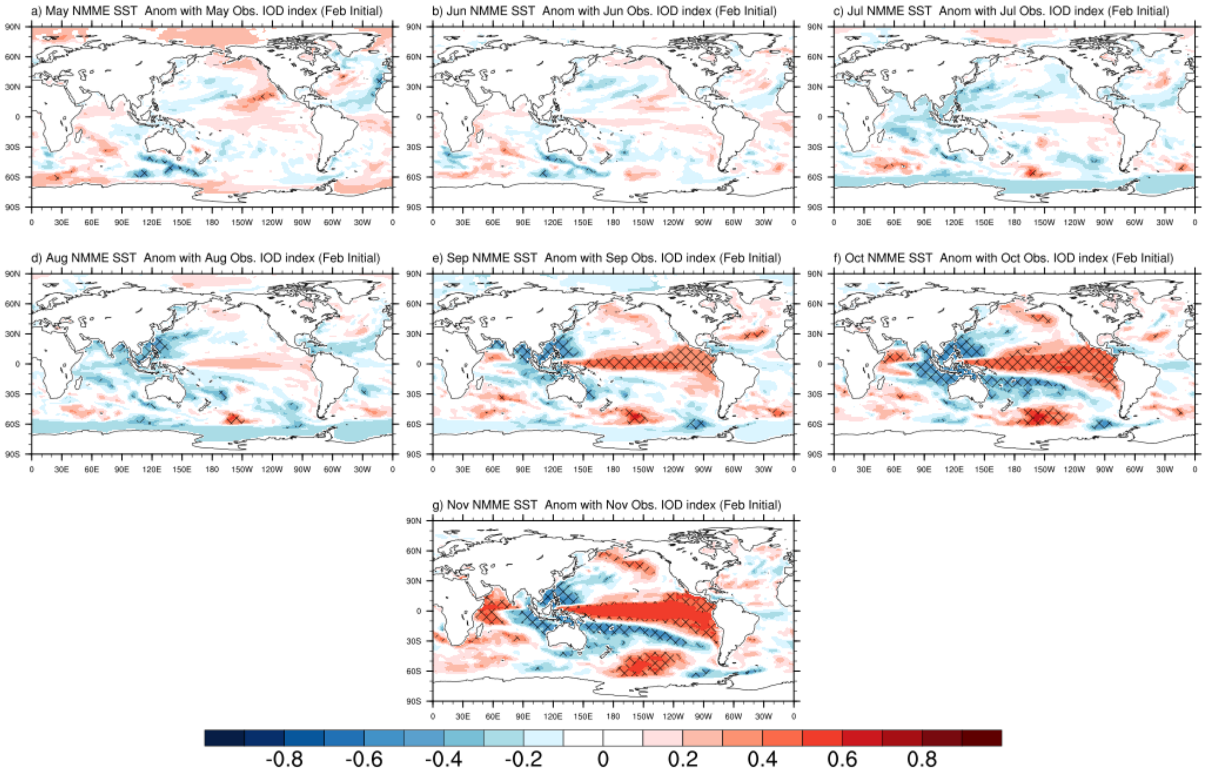

Fig S28. Spatial distribution of correlation coefficients of observed IOD index of May-November with ensemble mean May-November SST anomalies of February initialized NMME models for the period 1982-2018. The correlations significant at 95% using Student's two tailed test are marked with crosses.

The figure was prepared using The NCAR Command Language (version 6.4.0) [Software]. (2017). Boulder, Colorado: UCAR/NCAR/CISL/TDD. http://dx.doi.org/10.5065/D6WD3XH5

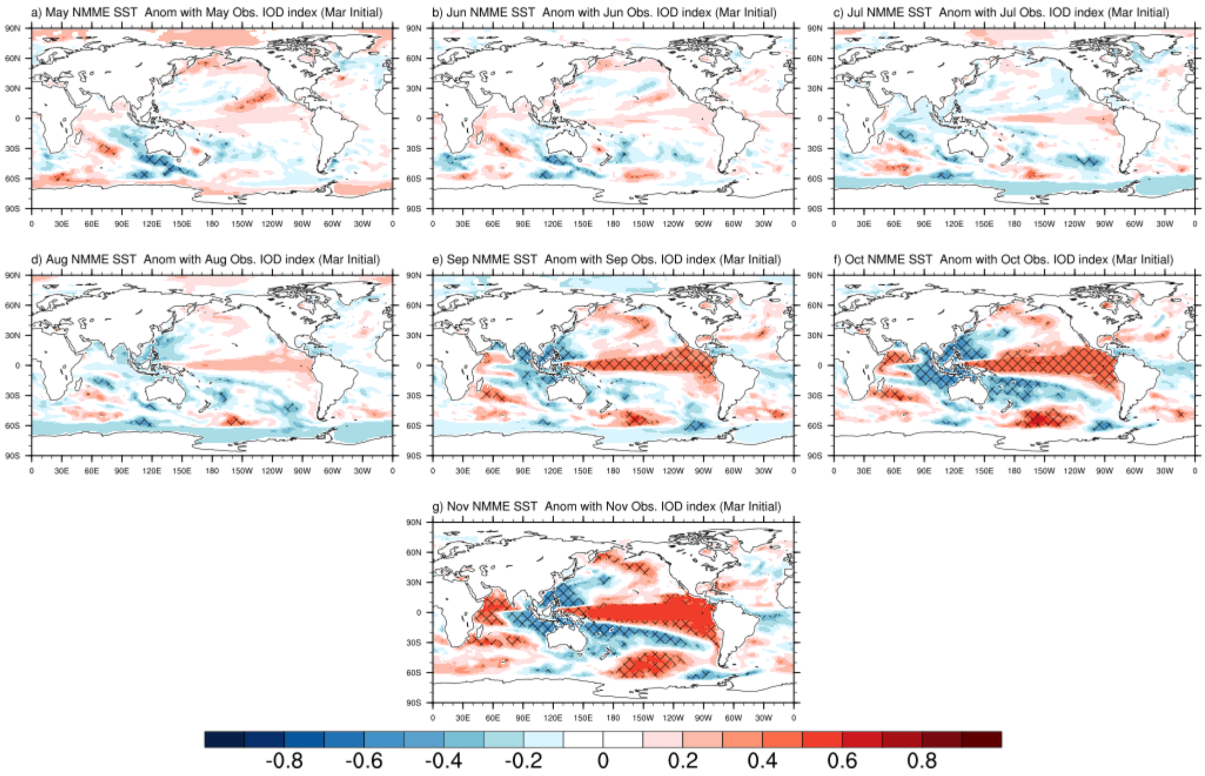

Fig S29. Spatial distribution of correlation coefficients of observed IOD index of May-November with ensemble mean May-November SST anomalies of March initialized NMME models for the period 1982-2018. The correlations significant at 95% using Student's two tailed test are marked with crosses.

The figure was prepared using The NCAR Command Language (version 6.4.0) [Software]. (2017). Boulder, Colorado: UCAR/NCAR/CISL/TDD. http://dx.doi.org/10.5065/D6WD3XH5

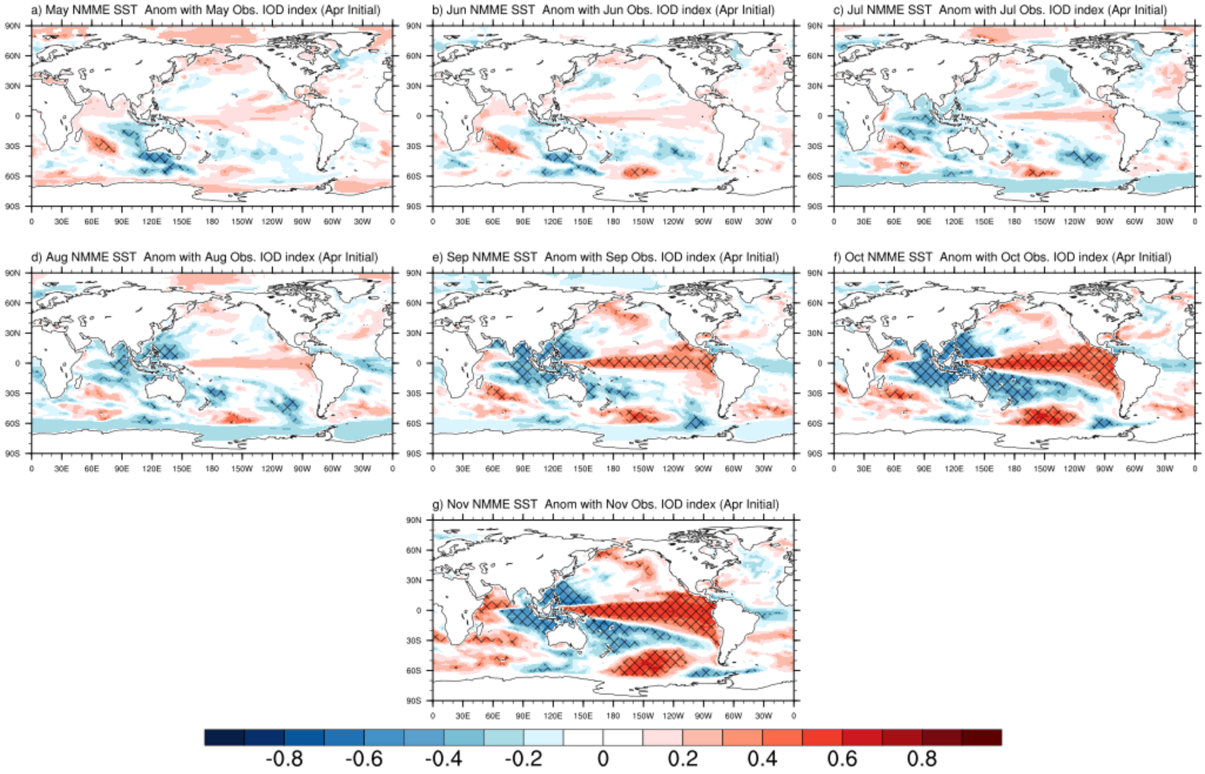

Fig S30. Spatial distribution of correlation coefficients of observed IOD index of May-November with ensemble mean May-November SST anomalies of April initialized NMME models for the period 1982-2018. The correlations significant at 95% using Student's two tailed test are marked with crosses.

The figure was prepared using The NCAR Command Language (version 6.4.0) [Software]. (2017). Boulder, Colorado: UCAR/NCAR/CISL/TDD. http://dx.doi.org/10.5065/D6WD3XH5
